# KCTD10 inhibits lung cancer metastasis and angiogenesis via ubiquitin-mediated β-catenin degradation

**DOI:** 10.3389/fimmu.2025.1630311

**Published:** 2025-08-12

**Authors:** Zihao Yin, Shengwen Long, Hao Zhou, Mi Ouyang, Qinghao Wang, Jun He, Rongyu Su, Zhiwei Li, Xiaofeng Ding, Shuanglin Xiang

**Affiliations:** ^1^ The National & Local Joint Engineering Laboratory of Animal Peptide Drug Development, College of Life Science, Hunan Normal University, Changsha, China; ^2^ State Key Laboratory of Developmental Biology of Freshwater Fish, College of Life Science, Hunan Normal University, Changsha, China; ^3^ Hunan Provincial Key Laboratory of Regional Hereditary Birth Defects Prevention and Control, Changsha Hospital for Maternal & Child Health Care Affiliated to Hunan Normal University, Changsha, China; ^4^ Institute of Interdisciplinary Studies, Hunan Normal University, Changsha, China; ^5^ Peptide and Small Molecule Drug R&D Platform, Furong Laboratory, Hunan Normal University, Changsha, China

**Keywords:** KCTD10, lung cancer metastasis, specific Kctd10 knockout, β-catenin, PD-1, M6A

## Abstract

Lung cancer remains a critical global health concern, characterized by the highest incidence and mortality rates among all cancers. Due to its heterogeneity and complexity, the molecular mechanism underlying lung cancer occurrence and progression needs to be further investigated. KCTD10 has been implicated in malignant phenotypes of several tumors, but the role of KCTD10 in lung cancer remains largely unexplored. In this study, we found that KCTD10 expression is significantly reduced in lung cancer tissues, and overexpression of KCTD10 could inhibit lung cancer progression both *in vitro* and *in vivo*. Immunoprecipitation-mass spectrometry (IP-MS), co-immunoprecipitation (Co-IP), and ubiquitination assays revealed that the BTB domain of KCTD10 interacts with Armadillo repeat domains 1–9 of β-catenin and facilitates ubiquitin-dependent degradation of β-catenin via the K48-linked ubiquitin chains, followed by the downregulation of the β-catenin downstream target gene PD-L1. Notably, the combined treatment of KCTD10 overexpression with anti-PD-1 antibodies exhibited a synergistic effect in suppressing lung cancer progression and brain metastatic colonization in mice. In addition, vascular endothelial cell-specific knockout of Kctd10 (Kctd10^flox/flox^CDH5^CreERT2/+^) promoted lung cancer metastasis and tumor angiogenesis through β-catenin signaling. Finally, we identified METTL14- mediated N6-methyladenosine (m^6^A) modification within the coding sequence (CDS) region of KCTD10, which enhanced KCTD10 mRNA stability in a YTHDF2-dependent manner. These findings highlight KCTD10 as a critical regulator of lung cancer progression and the tumor microenvironment, suggesting its potential as a promising therapeutic target for lung cancer.

## Introduction

Lung cancer is the most prevalent malignancy and the leading cause of cancer-related deaths worldwide, with approximately 85% of non-small cell lung cancer (NSCLC) (1). Despite advancements in treatment, lung cancer remains a major global health challenge (2, 3). Continued research into the molecular mechanisms underlying lung cancer progression and therapy resistance is crucial for the development of effective targeted therapies.

β-catenin is a key oncogenic driver in multiple cancers, including colorectal, breast, ovarian and gastric cancers (4–8), and has been reported to enhance lung cancer development (9). β-catenin can improve the expression of downstream genes such as ZEB1 and cyclin-D1, promoting tumor proliferation, metastasis and drug resistance (10, 11). Moreover, β-catenin also alters lung epithelial cell phenotypes through epigenetic modification, contributing to lung cancer progression (12, 13). Tumor metastasis is closely associated with epithelial-mesenchymal transformation (EMT), and the conversion of the epithelial phenotype to a mesenchymal phenotype enhances metastatic potential (14, 15). Studies have addressed that β-catenin activates EMT and induces metastasis in lung cancer, colorectal cancer and hepatocellular carcinoma (11, 16–19). In lung cancer, cancer-associated fibroblast-derived SDF-1 promotes EMT through β-catenin signaling (20). Additionally, β-catenin and Akt signaling pathways are critical for maintaining an EMT-associated cancer stem cell-like phenotype in breast and cervical cancers (21). Additionally, Wnt/β-catenin signaling contribute to immune evasion and resistance to immune checkpoint inhibitors in several cancers, including NSCLC (22). Specifically, β-catenin enhances PD-L1 transcription and upregulates PD-L1 expression in lung cancer (10). The EMT/β-catenin/STAT3/PD-L1 axis accumulates in cancer stem cells and drives immune escape in glioblastoma (23, 24). Targeting CBP/β-catenin in combination with PD-L1 blockade has emerged as a potential therapeutic strategy for colon cancer liver metastases (25). Moreover, downregulation of β-catenin prevents M2 macrophage-mediated angiogenesis in lung cancer (26). Therefore, β-catenin plays a critical role in EMT regulation and tumor immune evasion.

KCTD10, a member of the PDIP1 gene family encoding a potassium ion tetramer channel protein (27) has been involved in embryonic angiogenesis and cardiac development through negatively regulating Notch signaling (28, 29). Knockdown of KCTD10 reduces VEGF secretion and affects angiogenesis in diabetic retinopathy (30), suggesting that KCTD10 interferes with angiogenesis in both physiologic and pathologic angiogenic processes. KCTD10 has also been linked to the development of certain, including gastrointestinal stromal tumor (GIST) and pancreatic cancer (31–34). The cullin-3/KCTD10 E3 ubiquitin ligase complex promotes RhoB degradation, and activates epidermal growth factor (EGF)/human epidermal growth factor receptor 2 (HER2)-dependent Rac1 signaling in HER2-positive breast cancer cells (35). Conversely, in hepatocellular carcinoma (HCC), KCTD10 acts as a tumor suppressor by promoting p53 expression via Notch signaling (36), suggesting its context-dependent roles in tumor malignancy.

Although KCTD10 has been reported to interact with PCNA in A549 lung cancer cells (37), its precise function and molecular mechanisms in lung cancer are elusive. In this study, we demonstrated that KCTD10 suppresses lung cancer proliferation and metastasis by promoting β-catenin degradation, leading to decreasing PD-L1 expression and enhanced efficacy of anti-PD-1 immunotherapy in lung cancer and lung cancer brain metastases. Furthermore, endothelial-specific knockout of Kctd10 promotes lung cancer metastasis and angiogenesis. The stability of KCTD10 mRNA is enhanced by METTL14/YTHDF2-mediated m^6^A modification. Our findings establish KCTD10 as a critical regulator of both tumor progression and the tumor environment, highlighting the therapeutic potential of the novel METTL14/KCTD10/β-catenin regulatory axis in lung cancer treatment.

## Materials and methods

### Database analysis

Pan-cancer analysis was performed by UALCAN (https://ualcan.path.uab.edu/) (38). Gene expression, correlation analysis and survival analysis was from GEPIA (http://gepia.cancer-pku.cn/) (39). Survival analysis was from Kaplan-meier-plotter (https://kmplot.com/analysis/) (40). All survival analysis data were obtained from the Kaplan-meier-plotter website. The survival difference between groups was assessed using the log-rank test. Hazard ratios (HR), 95% confidence intervals (CI), and P-values were calculated using the Cox proportional hazards regression model. m^6^A site prediction was performed by SRAMP (http://www.cuilab.cn/sramp) (41).

### Cell culture and transfection

Authenticated A549, murine lewis lung cancer cells (LLC), H1437, Beas-2b, H446 and H460 cell lines (Institute of Biochemistry and Cell Biology, Chinese Academy of Sciences) were cultured in DMEM medium (Gibco, Gran Island, NY, USA). All these cells were cultured with 10% fetal calf serum (Gibco), 4 mM glutamine (Gibco), 100 U/ml penicillin and streptomycin (Invitrogen Life Technologies, Carlsbad, CA, USA) at 37°C in a 5% CO_2_ incubator. Cells were transfected with plasmid DNA and siRNAs using Lipofectamine 3000 (Invitrogen) according to the manufacturer’s instructions.

### Western blots

Cells were lysed with RIPA buffer (Beyotime, Shanghai, China), and protein extracts were separated on SDS-PAGE gels and transferred onto PVDF membranes (Bio-Rad, Richmond, CA) as previously described (42). Nuclear and cytoplasmic proteins were separated using a nuclear protein extraction kit (Solarbio, Beijing, China). Rabbit antibodies against KCTD10 (27279-1-AP, Proteintech, Wuhan, China) (1:1000), β-catenin (ET1601-5, HUABIO, Hangzhou, China) (1:1000), PD-L1 (ab228415, Abcam, Waltham, USA) (1:1000), E-cadherin (A3044, ABclonal, Wuhan, China) (1:1000), N-cadherin (A0433, ABclonal) (1:1000), METTL14 (ab309096, Abcam) (1:1000), YTHDF2 (ab220163, Abcam) (1:1000), Lamin B1 (A1910, ABclonal) (1:1000), Tubulin (AF7010, Affinity Biosciences, Changzhou, China) (1:5000) and Flag-Tag (F2555, Sigma) (1:1000) were used **Supplementary Table 1**. Mouse monoclonal anti-ubiquitin (sc-8017, Santa Cruz Biotech, Texas, USA) were used. HRP-conjugated goat anti-rabbit and goat anti-mouse secondary antibodies were from ABclonal. Western blots were independently replicated for at least three times.

### Immunohistochemical analysis and hematoxylin and eosin staining

Lung and brain tissues, along with corresponding tumor tissues were examined. Polyformalin-fixed paraffin-embedded (FFPE) tissues were processed through an alcohol gradient. Antigen retrieval was performed using citric acid solution (Service Biotechnology, Wuhan, China). Rabbit antibodies against KCTD10 (HPA014273, Sigma) (1:200, GAR), β-catenin (1:200), PD-L1 (1:200), c-Myc (380784, Zenbio, Chengdu, China) (1:200), VEGFR2 (A5609, ABclonal) (1:200), E-cadherin (1:200) and N-cadherin (1:200) were used. Mouse monoclonal antibodies against CD31 (ab9498, Abcam) (1:200), CD8α (70306, Cell Signaling, Massachusetts, USA) (1:200), Vimentin (240140, Zenbio) (1:200) were used. The primary antibodies were incubated overnight after blocking, and the HRP-conjugated goat anti-rabbit and goat anti-mouse secondary antibodies (Service Biotechnology) and DAB detection kit (Sevice Biotechnology) were incubated sequentially with the tissues, and the nuclei were counterstained with hematoxylin dye solution (Sevice Biotechnology). Sections were visualized under an Olympus BX53 microscope (Japan) after neutral resin sealing. The pathology information of human lung cancer tissues was shown in **Supplementary Tables 2** and **3**. HE staining was performed following standard protocols. These experiments were approved by the Human Ethics Committee of Hunan Normal University (2021–017).

### Plasmid construction

Full-length and truncated fragments of KCTD10 were cloned into pCMV-HA and pCMV-Myc vectors (Invitrogen), respectively. Full-length and deletion constructs of β-catenin were generated as described (43). The pcDNA3.1-(HA-Ub) was generated by inserting human UBC into pCDNA3.1-HA vector (Addgene, Massachusetts, USA). The HA-K0 and KCTD10 5′UTR was synthesized by Sangon Biotech. The K27R, K33R and K48R mutations were amplified from the pcDNA3.1-(HA-Ub) plasmid and the K27O, K33O and K48O mutations were amplified from the pcDNA3.1-(HA-K0) plasmid using splicing overlapping extension polymerase chain reaction (SOE-PCR) site-directed mutagenesis. DNA fragments containing K11R, K63R, K11O and K63O were digested with SalI and HpaII, and ligated to the pcDNA3.1-(HA-Ub) and pcDNA3.1-(HA-K0) digested with the same enzymes, respectively. The KCTD10 CDS, 3′UTR and 5′UTR were inserted into to the pGL3 reporter plasmid (Promega, MA, USA). The primers used are listed in **Supplementary Table 4**. All constructs were verified using the Sanger method (Sangon Biotech).

### Generation of KCTD10-overexpressing cell lines

KCTD10 lentivirus expression vectors and packaging plasmids (pHelper 1.0 and pHelper 2.0) (Genechem, Shanghai, China) were cotransfected into 293T cells, and viral supernatants were harvested, filtered, concentrated and titrated (44). The lentiviral expression vector GV365 included GFP protein and puromycin resistance gene for the observation of infection efficiency and screening of stable cell lines. Cells were placed in 6-well plates and infected at MOI=10 and observed for fluorescence four days post-infection. stable cell lines were selected with complete medium with 2 μg/ml puromycin (Solarbio), and maintained in medium with reduced puromycin concentration.

### Cell proliferation

For colony formation, 1,000 cells were placed per well in 6-well plates. Cells were cultured for two weeks. Cells were fixed with methanol, stained with 0.5% crystal violet, and counted. For MTT assays, 10, 000 cells were placed per well in 96-well plates, incubated with MTT reagent for 4 h and dissolved in DMSO. Absorbance at 492 nm was measured using a spectrophotometer (UV-2102C, Unico, Changsha, China).

### Cell migration and invasion assays

For wound healing assays, cells were cultured in 24-well plates until reaching 90% confluence. A 10-μl pipette tip was used to generate wounds. After wound generation, cells were changed to medium containing 2% serum. Three wound areas in each well were marked on the bottom of the plates and imaged at 0, 24 and 48 h after wound formation, which was photographed with the microscope at each time point. For cell migration, the chamber (Corning, New York, USA) was inserted into a 24-well plate, 2×10^4^ infected cells were distributed in the upper chamber with 10% or 15% FBS in the lower chamber. Cells were fixed, stained with 0.5% crystal violet (Sangon Biotech, Shanghai, China) and imaged under the microscope. Invasion assays were performed using the chamber covered with Matrigel glue (Corning), which were carried out as previously described (45).

### Mice

4-week-old nude mice were purchased from the Hunan SJA Laboratory Animal Corporation (Changsha, China). 6-week-old C57BL/6J mice were purchased from Jackson Laboratories (BarBarbor, ME). 5-week-old *CDH5* ^CreERT2/+^ and *KCTD10* ^flox/flox^ mice were obtained from GemPharmatech (Nanjing, China) (28) (**Supplementary Table 5**). Mice were maintained in a 12 h light/dark cycle and regularly fed with chow and water in an SPF room. All procedures were approved by Hunan Normal University (2021–017).

### Subcutaneous tumors and lung tumors of mice and tumor immunotherapy

1x10^7^ cells were injected subcutaneously into 4-week-old nude mice, with tumor growth measured twice weekly until tumor volume reached 1,000 mm^3^. For lung colonization assays, 5×10^5^ LLC cells were injected via the tail vein of 6-week-old C57BL/6J mice or 4-week-old nude mice, and lungs were removed after 4 weeks. For brain metastases, 5×10^5^ LLC cells were injected intracranially into 6-week-old C57BL/6J mice and brains were harvested 4 weeks after injection as previously described (46). Anti-PD-1 therapy (100 µg/mouse, RPM1-14, BioXCell, New Hampshire, USA) was injected intraperitoneally on days 11/14/17/20 in LLC models. Tumor weight, volume, survival and tissue analysis were recorded and analyzed. All mouse experiments were repeated at least twice.

### Immunoprecipitation and mass spectrometry

Cells in 10 cm dishes were grown to 80% confluence and transfected with 5 μg *KCTD10* and *β-catenin* (full length or truncated). After 30 h, cells were lysed and whole cell extracts were immunoprecipitated using rabbit polyclonal antibodies against Myc-tag (C3956, Sigma) or HA-tag (05-904, Sigma) and protein A/G plus beads (K1305, APEXBIO, Texas, USA). Immunoprecipitates were resolved by 10% SDS-polyacrylamide gels and detected by HA-tag or Myc-tag. Rabbit preimmune IgG (ab37355, Abcam) served as a negative control. For mass spectrometry, proteins from KCTD10-overexpressing A549 cells were separated in 10% SDS-PAGE gels after immunoprecipitation using either IgG or Flag-tag (F7425, Sigma), and stained using a Protein Fast Silver Stain Kit (Leagene Biotechnology, China) and analyzed by mass spectrometry (Novogene, Beijing, China) (47, 48).

### Protein degradation and ubiquitination assays

To assess protein stability, cells and KCTD10-overexpressing cells were treated with 50 μg/mL CHX (Selleck Chemicals, Texas, USA) for 0, 2, 8 h, respectively. The proteins were extracted and detected through Western blotting. To identify protein degradation pathway, transfected cells were treated with 20 µM MG132 for 10 h before harvesting. Cells were lysed and analyzed by Western blotting with antibodies against Flag-Tag, β-catenin and Tubulin. For ubiquitin experiments, cells were transfected either with the expression plasmids pCMV-Myc-β-catenin alone or with pCMV-HA-KCTD10 and pcDNA3.1-(HA-Ub) or its mutants. 24 h after transfection. Myc-β-catenin was immunoprecipitated with rabbit polyclonal anti-β-catenin antibodies and these immunoprecipitates were subjected to Western blotting with ubiquitin to detect β-catenin-ubiquitin conjugation.

### Immunofluorescence double staining

Tissue sections were dehydrated and processed for antigen retrieval. For cell immunofluorescence double labeling, the cells were fixed with methanol. The tissue sections and cells were blocked and incubated overnight with the first primary antibodies, followed by incubation with the specific secondary antibodies. After antigen repair, the second primary antibodies were added for overnight incubation, and another species-specific secondary antibodies were added for incubation. Primary antibodies are KCTD10 (1:100), β-catenin (1:100), CD31 (1:200), α-SMA (250104, Zenbio) (1:100). Alexa Fluor 488 phalloidin goat anti-rabbit (A-11008, Thermo Fisher Scientific, Massachusetts, USA) (1:2000) and Alexa 594 goat anti-mouse antibodies (A11005, Thermo Fisher Scientific) (1:2000) were used as secondary antibodies. The nucleus was stained with Hoechst 33258 (Beyotime). The fluorescence signals were analyzed with a fluorescence microscope (Zeiss Axioskop-2).

### Generation of endothelial cell-specific Kctd10 knockout mice and lung tumor model

Kctd10^flox/flox^ and CDH5^CreERT2/+^ mice were intercrossed and screened to generate homozygous Kctd10^flox/flox^CDH5^CreERT2/+^ mice. To obtain the CDH5^CreERT2/+^Kctd10^-/-^ mice, the mice were administered intraperitoneally with tamoxifen (75 mg/kg body weight) for one week. For the LLC mouse models, 1×10^6^ tumor cells were injected via the tail vein into tamoxifen-injected 6-week-old female Kctd10^flox/flox^ mice or CDH5^CreERT2/+^ Kctd10^-/-^mice.

### RNA extraction and qRT-PCR

Total RNA was extracted from the cell lines using TRIzol reagent (Thermo Fisher Scientific) and reverse transcribed into cDNA using MMLV RTase and random primers (Sangon Biotech). SYBR green (Invitrogen)-based real-time PCR was performed using an ABI 7900 thermocycler (Thermo Fisher Scientific). Reactions were incubated in a 96-well plate at 95°C for 5 min, followed by 35 cycles of 95°C for 20 sec and 60°C for 30 sec. The PCR primers are listed in **Supplementary Table 4**. The relative expression levels of genes were calculated by the 2^−ΔΔCt^ method compared to β-actin.

### m^6^A assay

m^6^A modification sites were predicted on the SRAMP website. For MeRIP assays, RNA was isolated and incubated with 2 μg m^6^A (A19841, ABclonal) or IgG antibodies. Precipitated RNA was reverse transcribed, amplified and then subjected to agarose gel electrophoresis as described (49). m^6^A-related siRNAs were purchased from genepharma (Shanghai, China) (**Supplementary Table 6**).

### Luciferase reporter assays

Cells were co-transfected with recombinant pGL3 vectors bearing CDS, 3′UTR and 5′UTR of the KCTD10 gene or specific siRNAs. After the transfection, β-galactosidase and luciferase activities were measured using the Luciferase Assay System (Promega, Madison, WI) in a TD-20/20 luminometer (Turner Design, Sunnyvale, CA) as previously reported (36).

### Statistical analysis

Data are expressed as mean ± SD of at least three independent experiments. Statistical analysis was performed using GraphPad Prism 7 (San Diego, California, USA) and SPSS 22.0 (SPSS Inc., Chicago, Illinois, USA). The significance of the differences between groups was determined using Student’s *t*-test and multi-group comparisons was determined using one-way ANOVA with *post hoc* tests. Values of P<0.05 were considered statistically significant. *p < 0.05, **p < 0.01, ***p < 0.001.

## Results

### KCTD10 expression is downregulated in lung cancer tissues and correlates with favorable patient prognosis

**Figure 1:**
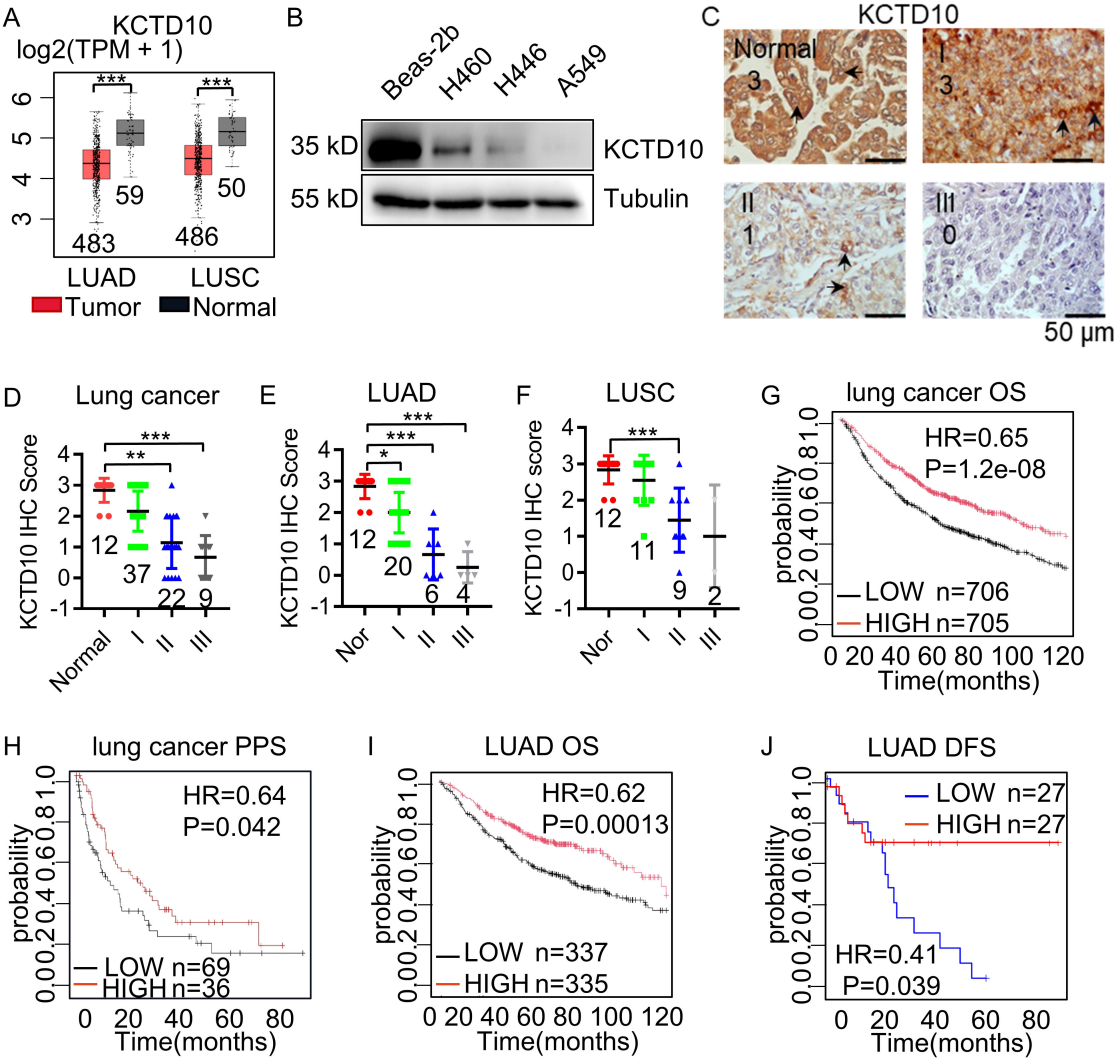
Low expression of KCTD10 in lung cancer. **(A)** GEPIA database analysis of KCTD10 expression in normal lung tissues and lung cancer tissues. **(B)** Western blot analysis of KCTD10 expression in lung cancer cell lines and normal cell line Bears-2b. **(C, D)** IHC analysis of KCTD10 expression in human lung cancer tissues (n=80) and corresponding staining scores in different lung cancer grades. **(E, F)** IHC analysis of KCTD10 expression in LUAD (n=42) and LUSC (n=34). **(G)** Correlation between KCTD10 expression and overall survival in lung cancer patients, HR=0.65 (0.55-0.75), logrank P=1.2e-08. **(H)** Correlation between KCTD10 expression and post-progression survival of lung cancer patients, HR=0.64 (0.42-0.99), logrank P=0.042. **(I)** Correlation between KCTD10 expression and overall survival in LUAD patients, HR=0.62 (0.48-0.79), logrank P=0.00013. **(J)** Correlation between KCTD10 expression and disease free survival in LUAD patients, HR=0.41 (0.35-0.47), logrank P=0.042. *P < 0.05, **P < 0.01, ***P < 0.001.

To assess the expression of KCTD10 in lung cancer, we analyzed the TCGA Pan-Cancer and GEPIA database and found lower KCTD10 expression in lung cancer tissues than in normal tissues (**Figure 1A**, **Supplementary Figure 1A**). Western blots further confirmed reduced KCTD10 expression in lung cancer cell lines compared with bronchial epithelial Bears-2b cells (**Figure 1B**). IHC analysis revealed that KCTD10 expression was markedly lower in high-stage lung cancer patients (**Figures 1C, D**), with a particularly pronounced reduction in LUAD (**Figures 1E, F**). Kaplan-Meier Plotter survival analysis revealed that patients with high expression of KCTD10 exhibited significantly prolonger overall survival (OS) and post-progression survival (PPS) (**Figures 1G, H**), suggesting a potential tumor-suppressive role of KCTD10. Interestingly, the hazard ratio (HR) for KCTD10 was lower in LUAD, implying a notable role for KCTD10 in this lung cancer type (**Figure 1I**). As demonstrated in the GEPIA database analysis, elevated KCTD10 expression correlates with prolonged disease-free survival (DFS) in patients, suggesting its potential role in inhibiting tumor metastasis (**Figure 1J**). Thus, KCTD10 expression is inversely correlated with lung cancer stage and is associated with a favorable prognosis for lung cancer patients.

### Overexpression of KCTD10 suppresses lung cancer growth and metastasis

**Figure 2:**
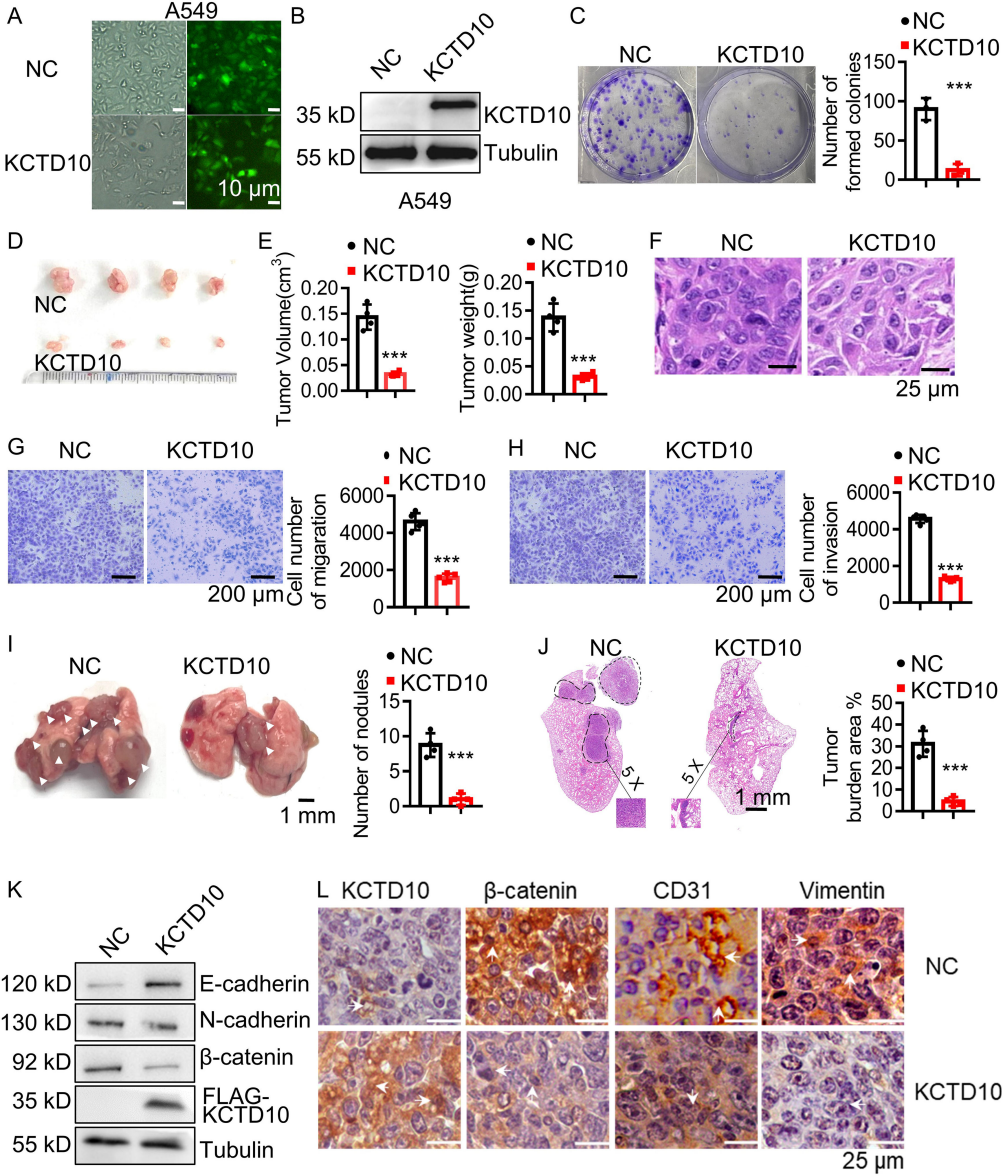
Overexpression of KCTD10 inhibits lung cancer growth and metastasis *in vitro* and *in vivo*. **(A)** Fluorescence image showing the efficiency of lentiviral infection in A549 cells. **(B)** Western blot analysis confirming KCTD10 overexpression in A549 cells. **(C)** Colony formation assays demonstrating the effect of KCTD10 overexpression on cell growth. **(D, E)** Effects of KCTD10 on the weight and volume of subcutaneous A549 tumors (n=4/group). **(F)** Effects of KCTD10 on cell morphology of A549 subcutaneous tumors. **(G, H)** Transwell assays evaluating the effect of KCTD10 overexpression on A549 cell migration and invasion. **(I, J)** Effect of KCTD10 overexpression on lung colonization of A549 cells injected via the tail vein (n=4). 5x10^5^ A549 cells and KCTD10-overexpressing A549 cells were injected. **(K, L)** Western blot and IHC analysis of EMT-related gene expression. ***P< 0.001.

To further investigate the functional role of KCTD10, we constructed a stable A549 lung cancer cell line overexpressing KCTD10 by lentiviral transduction and demonstrated successful overexpression of KCTD10 by fluorescence imaging and Western blots (**Figures 2A, B**, **Supplementary Figure 1B**). MTT assays revealed that overexpression of KCTD10 reduced cell viability while enhancing cisplatin-induced cytotoxicity (**Supplementary Figure 1C**). And colony formation assays further supported the suppressive effect of KCTD10 on the growth of A549 cells (**Figure 2C**). Subsequently, subcutaneous tumorigenesis assays showed that KCTD10-overexpression A549 cells formed significantly smaller tumors compared to controls (**Figures 2D, E**, **Supplementary Figure 1D**). HE staining revealed decreased tumor cell density in Kctd10-overexpressing tumors (**Figure 2F**, **Supplementary Figure 1E**). These results indicate that KCTD10 inhibits lung cancer growth both *in vitro* and *in vivo*.

Lung cancer metastasis remains a major clinical challenge (50). To determine whether KCTD10 influences metastatic ability of lung cancer, we performed wound healing, cell migration and invasion assays and found that KCTD10 overexpression significantly inhibited the migration and invasion ability of A549 cells (**Figures 2G, H**, **Supplementary Figure 1F**). In an *in vivo* metastasis model, tail vein injection of KCTD10-overexpressing A549 cells into nude mice resulted in a significant reduction in lung nodule formation (**Figure 2I**). HE staining further confirmed a lower lung tumor burden in the KCTD10-overexpressing group compared to the NC group (**Figure 2J**). Since tumor metastasis is closely linked to an EMT process (19), we examined EMT markers in A549 cells and subcutaneous tumors. Western blots and IHC analyses showed that overexpression of KCTD10 increased the epithelial marker E-cadherin while decreased the stromal markers N-cadherin and β-catenin (**Figures 2K, L**, **Supplementary Figures 2A, B**). Moreover, KCTD10-overexpressing cells exhibited a morphology resembling epithelial cells (**Supplementary Figure 2C**). Additionally, CD31 expression, an angiogenesis marker, was downregulated in KCTD10-overexpressing subcutaneous tumors, indicating reduced tumor angiogenesis (**Figure 2L**). Similar results were observed in LLC and H1437 cells (**Supplementary Figures 3A–F**). These findings suggest that KCTD10 inhibits lung cancer metastasis by suppressing EMT and tumor angiogenesis.

### KCTD10 interacts with β-catenin and promotes its ubiquitin-dependent degradation via the K48 ubiquitin chain

**Figure 3:**
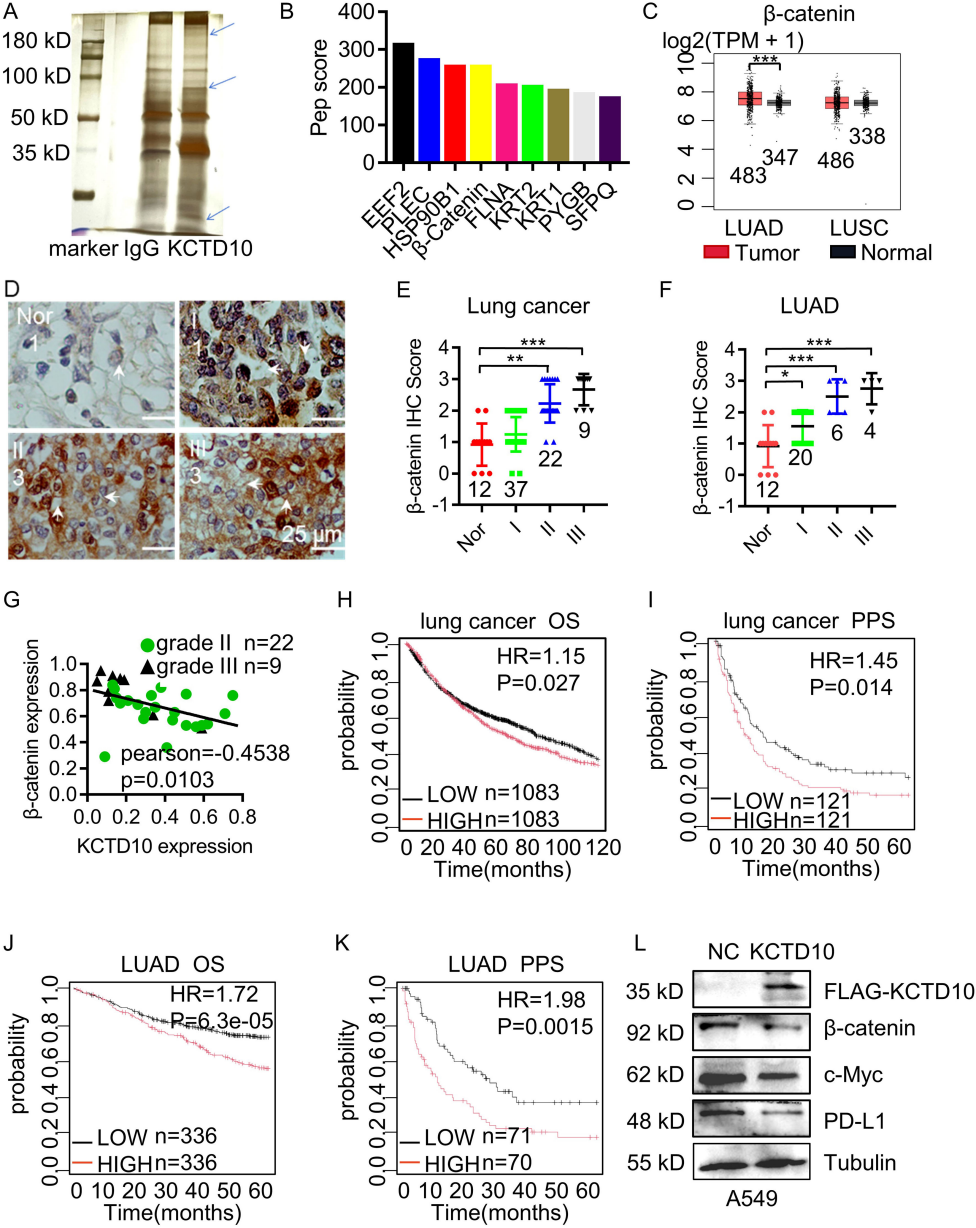
Interaction between KCTD10 and β-catenin proteins. **(A)** IP-MS and silver staining showing differential bands between anti-KCTD10 antibodies and IgG. **(B)** The top nine polypeptide scores of KCTD10-interacting proteins. **(C)** GEPIA database analysis of β-catenin expression in normal and lung cancer tissues. **(D, E)** IHC analysis of β-catenin expression and corresponding staining scores in different lung cancer grades (n=80). **(F)** IHC analysis of β-catenin in LUAD (n=42). **(G)** Correlation between KCTD10 and β-catenin expression in lung cancer. **(H–K)** Correlation between β-catenin expression and overall/post-progression survival of lung cancer patients. **(H)** HR=1.15 (1.02-1.29), logrank P=0.027. **(I)** HR=1.45 (1.08-1.97), logrank P=0.014. **(J)** HR=1.72(1.31-2.25), logrank P=6.3e-05. **(K)** HR=1.98 (1.29-3.05), logrank P=0.0015. **(L)** Western blot analysis of β-catenin expression and its downstream genes in A549 cells. *P < 0.05, **P < 0.01, ***P < 0.001.

To determine the molecular mechanism of KCTD10 in lung cancer, we performed IP followed by silver staining and MS. The identified differential bands revealed potential interacting KCTD10-interacting proteins (**Figure 3A**) and their predicted subcellular localization (**Supplementary Figure 4A**). KEGG enrichment analysis indicated strong associations between KCTD10-interacting proteins and metabolism, cancers and immune system (**Supplementary Figure 4B**). GO enrichment confirmed that KCTD10 highly correlated with protein binding, as previous reported (**Supplementary Figure 5A**) (51). KOGs analysis highlighted its involvement in post-translational modifications, protein turnover, chaperones and signal transduction (**Supplementary Figure 5B**). Based on protein-peptide scores, we identified β-catenin as a top interactor of KCTD10 (**Figure 3B**). β-catenin is well-established oncogene implicated in EMT and tumor progression (22). GEPIA database analysis showed that β-catenin is highly expressed in LUAD (**Figure 3C**). IHC analysis further showed a positive correlation between β-catenin expression and lung cancer stage (**Figures 3D, E**), particularly in LUAD (**Figure 3F**). The IHC scores revealed a negative correlation between KCTD10 and β-catenin expression (**Figure 3G**). Moreover, Kaplan-Meier survival analysis indicated that high β-catenin expression is associated with poor prognosis (**Figures 3H–K**). Importantly, overexpression of KCTD10 reduced β-catenin and its downstream effector, PD-L1, in lung cancer cells (**Figure 3L**, **Supplementary Figure 6**).

**Figure 4:**
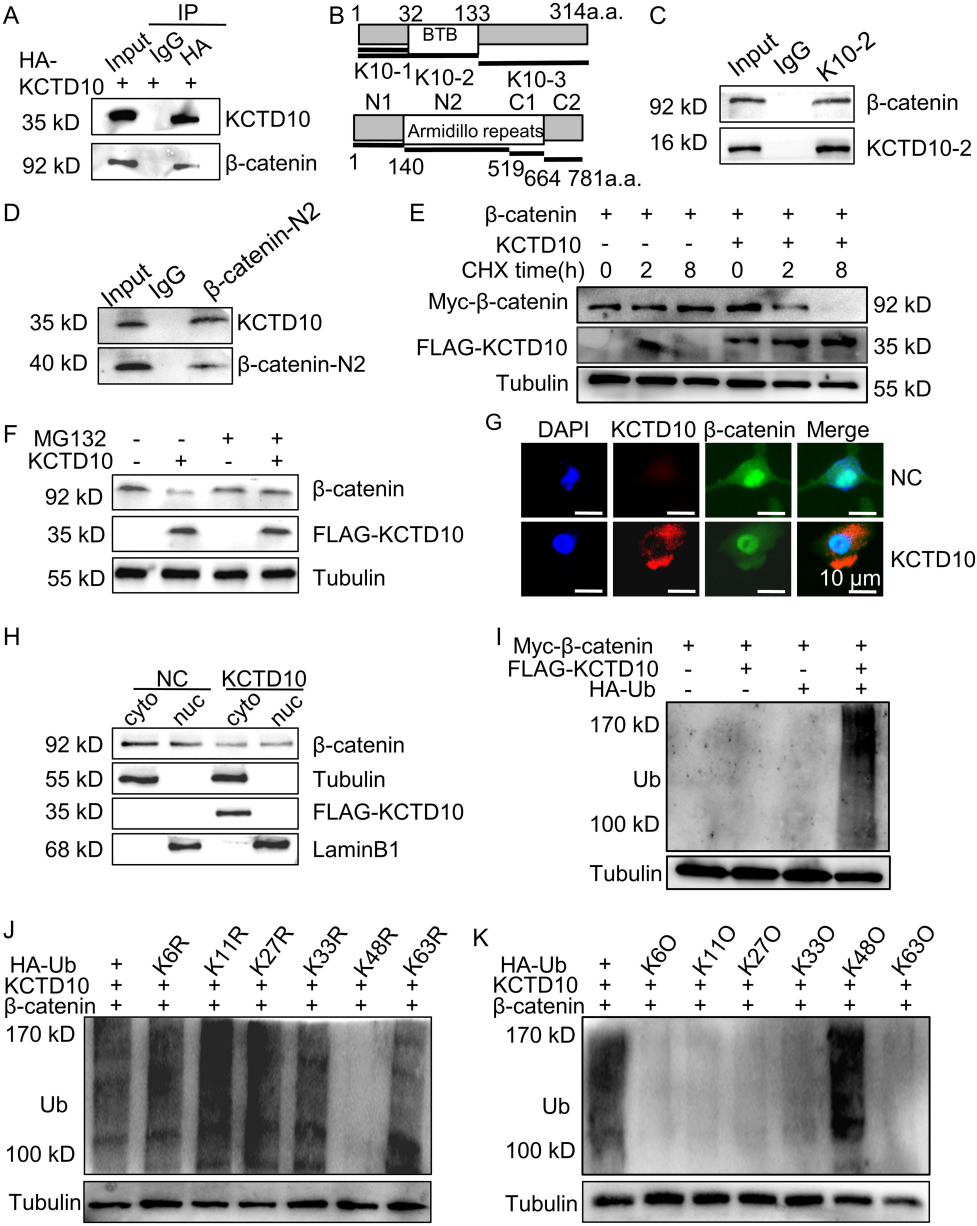
KCTD10 promotes ubiquitination and degradation of β-catenin through the K48-ubiquitin chain. **(A)** Co-IP analysis demonstrating the interaction between KCTD10 and β-catenin proteins. **(B)** Representative schematic of KCTD10 and β-catenin protein domains. **(C, D)** Identification of the interacting regions between truncated KCTD10 and β-catenin proteins. **(E)** Degradation of β-catenin proteins after CHX treatment. **(F)** Effect of KCTD10 on β-catenin protein stability in the presence of MG132. **(G, H)** Fluorescence analysis and Western blots showing KCTD10-induced degradation of cytoplasmic β-catenin. **(I–K)** KCTD10 overexpression enhanced the ubiquitination of β-catenin. Myc-β-catenin was immunoprecipitated with rabbit polyclonal anti-β-catenin antibodies and these immunocomplexes were subjected to Western blotting with anti-ubiquitin antibodies to detect β-catenin-ubiquitin conjugates. In the ubiquitin constructs, R indicates that the corresponding lysine residue has been mutated to arginine, abolishing linkage at that site; O indicates that only the corresponding lysine residue remains intact, while all other lysines are mutated, allowing selective assessment of linkage through that specific site.

To verify the interaction between KCTD10 and β-catenin, co-IP assays were performed. Endogenous β-catenin was detected in immune complexes of HA-KCTD10, whereas control IgG failed to precipitate any band (**Figure 4A**). To map the binding regions, we constructed truncated plasmids of KCTD10 and β-catenin, we found that the BTB region of KCTD10 binds to the Armadillo repeat region (1–9) of β-catenin (**Figures 4B–D**, **Supplementary Figures 7A–C**). We next examined whether KCTD10 promoted the degradation of β-catenin using cycloheximide (CHX), which revealed accelerated β-catenin degradation in KCTD10-overexpressing cells (**Figure 4E**), and this was rescued by the proteasome inhibitor MG132 (**Figure 4F**), suggesting KCTD10 mediates β-catenin degradation via the ubiquitin-proteasomal pathway. Immunofluorescence and nucleocytoplasmic fractionation experiments confirmed that KCTD10 primarily degrades β-catenin in the cytoplasm (**Figures 4G, H**). Ubiquitination assays demonstrated that co-transfection of KCTD10 and ubiquitin plasmids promoted the ubiquitination of β-catenin (**Figure 4I**). To identify the specific polyubiquitin linkage, we constructed a series of lysine-linked ubiquitin active and mutant site plasmids and found that KCTD10 facilitates polyubiquitination of β-catenin via K48-linked ubiquitin chains (**Figures 4J, K**), supporting its role in the ubiquitin-proteasomal degradation of β-catenin. Similar results were observed in LLC and H1437 cells (**Supplementary Figures 8A-D**). Taken together, our results demonstrated that KCTD10 directly binds with β-catenin and promotes its K48-linked polyubiquitination and proteasomal degradation.

### Overexpression of KCTD10 enhances the therapeutic effect of PD-1 blockade in a metastatic lung cancer model

**Figure 5:**
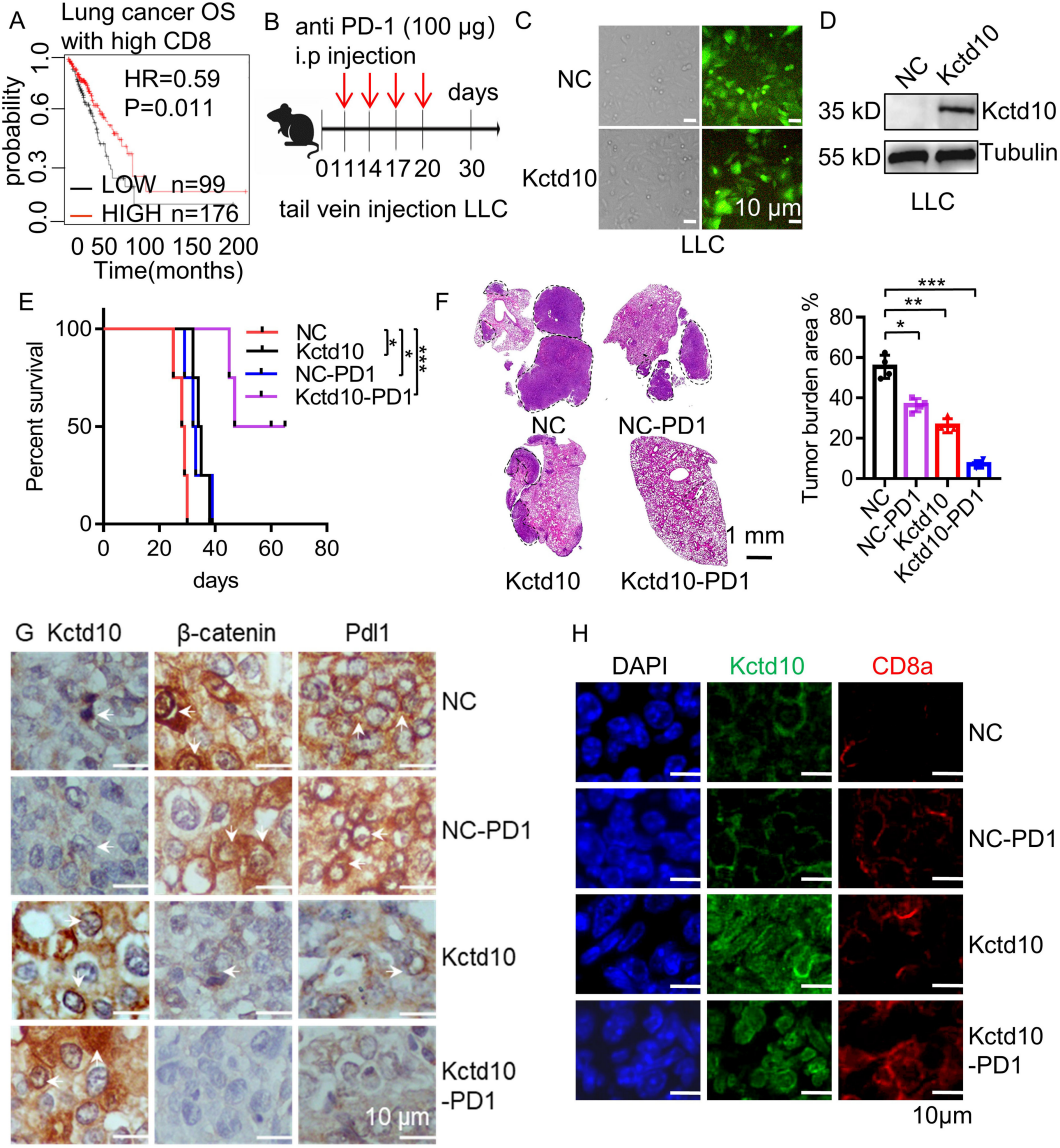
Overexpression of Kctd10 combined with anti-PD-1 therapy effectively suppresses lung tumor colonization. **(A)** Effect of Kctd10 expression on patient survival in high CD8 expression cohorts. **(B)** Combined therapeutic strategy for LLC tumors inoculated into C57BL/6 mice. **(C, D)** Construction and validation of stable LLC cell lines overexpressing Kctd10. **(E, F)** Effect of Kctd10 and anti-PD-1 therapy on lung tumor size and survival in LLC-bearing mice (n=4/group). **(G)** IHC analysis of β-catenin and Pd-l1 proteins expression following Kctd10 overexpression and anti-PD-1 treatment. **(H)** IF analysis of CD8a proteins expression following Kctd10 overexpression and anti-PD-1 treatment. *P < 0.05, **P < 0.01, ***P < 0.001.

Since KCTD10 downregulates β-catenin and PD-L1, and PD-L1 expression is known to promote tumor immune evasion in lung cancer (52), we investigated the impact of KCTD10 on lung cancer immunotherapy. Kaplan-Meier Plotter survival analysis revealed that high KCTD10 expression significantly correlated with improved prognosis in lung cancer patients with CD8^+^ T cell infiltration (**Figure 5A**, **Supplementary Figure 9A**), indicating a potential role in immune system activation. To assess the therapeutic potential of KCTD10 in combination with immune checkpoint blockade, KCTD10-overexpressing LLC cells were injected into 6 week-old C57BL/6J mice, followed by anti-PD-1 antibody treatment (**Figure 5B**). Fluorescence analysis and Western blots confirmed successful Kctd10 overexpression (**Figures 5C, D**). Survival analysis demonstrated that both Kctd10 overexpression and anti-PD-1 treatment individually prolonged the survival of C57BL/6 mice compared to the NC group. Notably, the combination of KCTD10 overexpression and anti-PD-1 therapy exhibited an additive effect, with 50% of the combination group surviving beyond 50 days, compared to fewer than 30 days in the NC group (**Figure 5E**). HE staining further showed that Kctd10 overexpression and PD-1 blockade reduced lung tumor burden, independently, with the combination treatment exerting the most pronounced effect (**Figure 5F**). IHC and IF analysis indicated that Kctd10 overexpression suppressed β-catenin and Pd-l1 expression while improved CD8a^+^ T cell infiltration. The combined treatment led to the most strongly improvement in CD8a^+^ T cell infiltration (**Figures 5G, H**, **Supplementary Figures 9B, C**). Therefore, overexpression of Kctd10, in conjunction with PD-1 blockade, effectively inhibits lung tumor metastasis and augments anti-tumor immunity.

**Figure 6:**
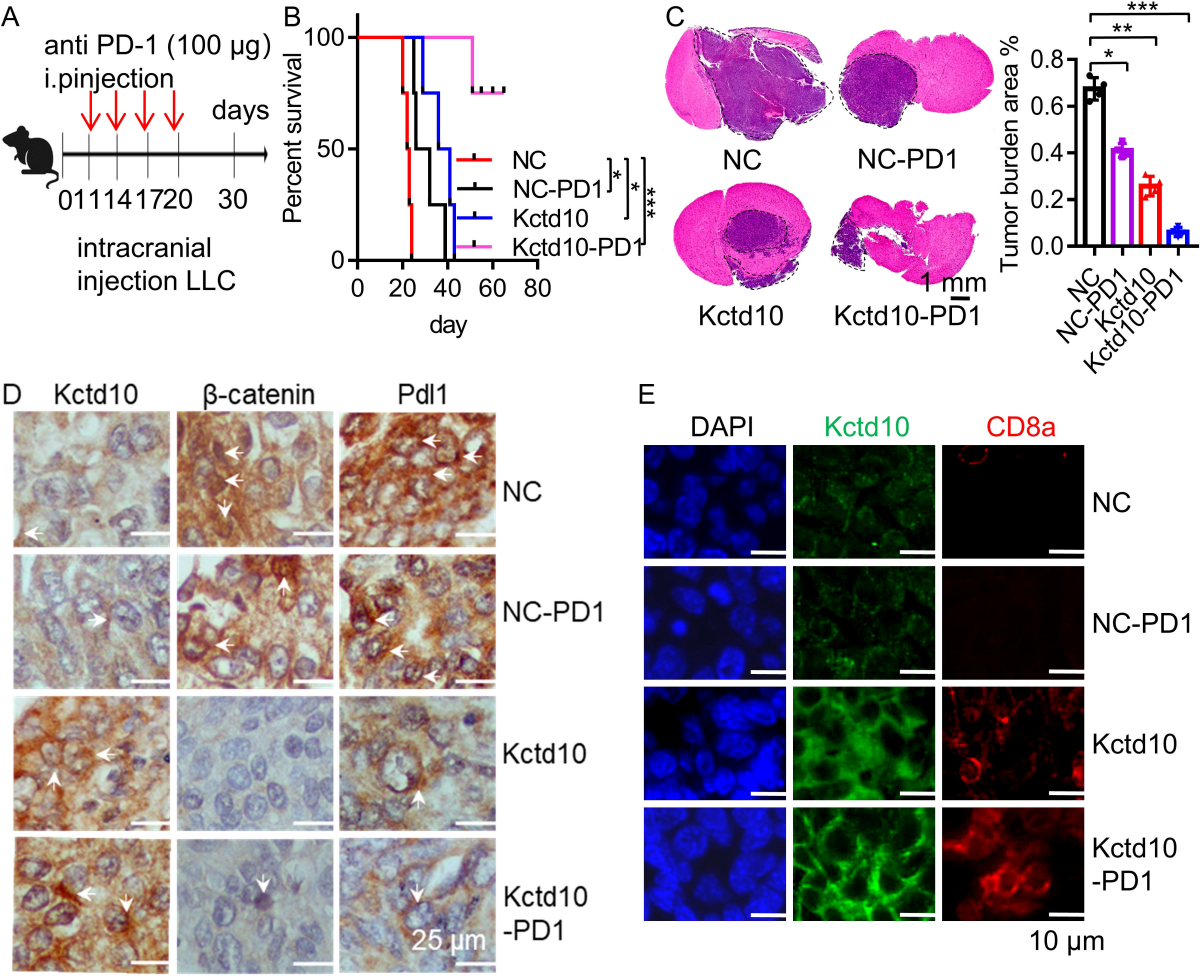
Kctd10 in combination with anti-PD-1 therapy suppressed lung cancer brain metastasis. **(A)** Combined strategy for intracranial LLC metastatic tumor treatment in C57BL/6 mice. **(B, C)** Effects of Kctd10 and anti-PD-1 therapy on intracranial tumor sizes and survival in LLC-bearing mice (n=4/group). **(D)** IHC analysis of β-catenin and Pd-l1 proteins following Kctd10 overexpression and anti-PD-1 therapy. **(E)** IF analysis of CD8a proteins following Kctd10 overexpression and anti-PD-1 therapy. *P < 0.05, **P < 0.01, ***P < 0.001.

Brain metastases are among the most common distant metastases in lung cancer (53). To investigate the effect of Kctd10 on brain colonization, we intracranially injected LLC cells into 6 week-old C57BL/6J mice and assessed the impact of Kctd10 overexpression combined with anti-PD-1 blockade (**Figure 6A**). Both Kctd10 overexpression and anti-PD-1 treatment independently prolonged the mouse survival compared to the NC group. Remarkedly, the combination therapy showed the greatest survival benefic, with 80% of the mice in this group survived beyond 60 days, whereas those in the NC group die at approximately 23 days (**Figure 6B**). HE staining revealed that both Kctd10 overexpression and anti-PD-1 treatment independently reduced brain tumor burden, with the combined treatment exhibiting the most profound effect (**Figure 6C**). IHC analysis of brain sections revealed that overexpression of Kctd10 downregulated β-catenin and Pd-l1 but increased CD8a^+^ T cell abundance, indicating the similar effects in the lungs. Notably, the combined therapy led to the most substantial increase in CD8a^+^ T cell infiltration, further enhancing the anti-tumor immune response (**Figures 6D, E**, **Supplementary Figures 9D, E**). These results highlight the therapeutic potential of KCTD10 overexpression in improving the efficacy of immune checkpoint blockade for lung cancer metastases.

### Endothelial-specific knockout of Kctd10 promotes tumor metastasis and angiogenesis in lung cancer

**Figure 7:**
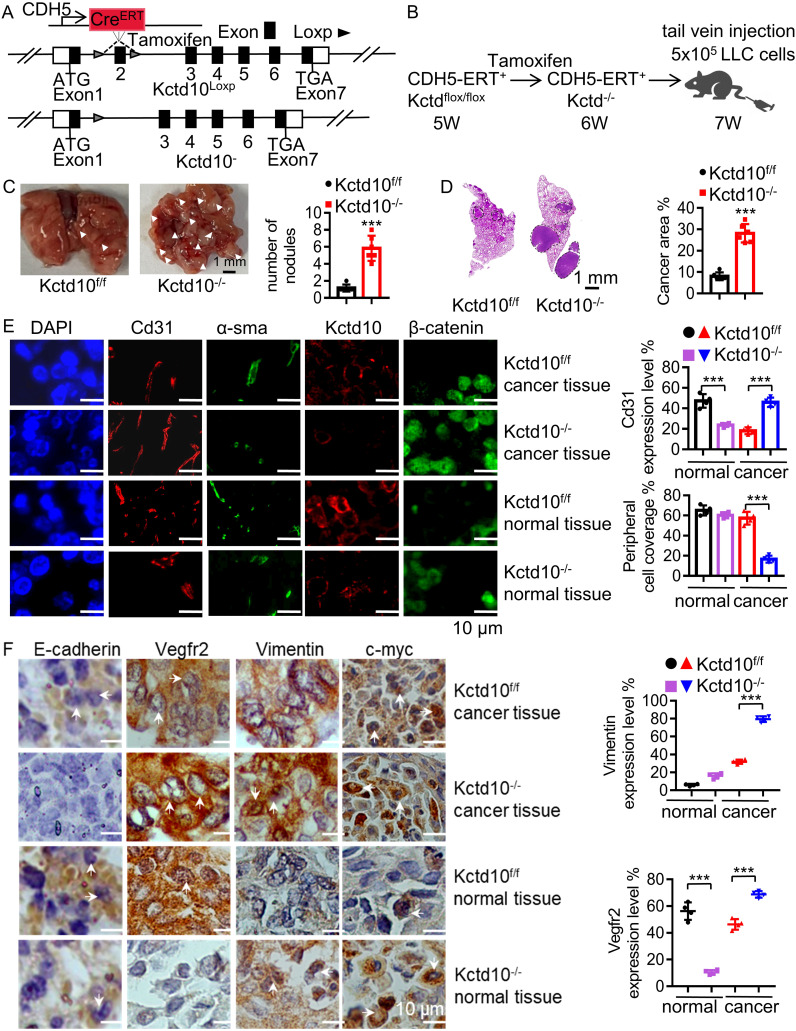
Endothelial Kctd10 knockout inhibits angiogenesis and metastatic phenotypes in lung tumors. **(A)** Construction strategy for Kctd10^flox/flox^CDH5^CreERT2/+^ mice. **(B)** Experimental strategy for inducible Kctd10 knockout and LLC cell injection in Kctd10^flox/flox^CDH5^CreERT2/+^ mice. Mice were treated by tamoxifen (75 mg/kg) for one week (n=5/group). **(C, D)** Images of lung cancer and corresponding HE staining in conditional knockout mice. **(E, F)** Immunofluorescence and IHC analysis of target gene expression in mouse lungs and lung tumor tissues. ***P < 0.001.

Tumor angiogenesis is critical for tumor growth and metastasis, while β-catenin can promote tumor angiogenesis via VEGF signaling (54). Immunofluorescence analysis showed that Kctd10 is co-localized with endothelial markers Cd31 and Cd34 in multiple subcutaneous tumors (**Supplementary Figures 10A–F**), suggesting that Kctd10 is associated with tumor angiogenesis. Given that endothelial cells in the tumor microenvironment are dispensable for tumor angiogenesis (55), we generated *Kctd10* ^flox/flox^ *CDH5* ^CreERT2/+^ mice that specifically delete Kctd10 in vascular endothelial cells (**Figures 7A, B**, **Supplementary Figure 10G**). Following injection of 1x10^6^ LLC cells (**Figure 7B**), we observed significantly increased lung tumor burden in Kctd10-knockout mice (**Figure 7C**). HE staining confirmed a large lung tumor area in these mice compared to controls (**Figure 7D**). Immunofluorescence analysis revealed a decrease in normal blood vessels, as indicated by Cd31 labeling, whereas the number of tumor-associated blood vessels was markedly increased in *Kctd10*-knockout mice (**Figure 7E**). Normal vessels have a higher pericyte density than tumor vessels (56). Additionally, pericyte coverage, assessed by α-sma staining, was reduced in tumor regions, indicating a shift toward an abnormal tumor vasculature phenotype. And the expression of β-catenin was upregulated in *Kctd10* knockout mice (**Figure 7E**, **Supplementary Figures 10H**, **11A**). The IHC results showed that the expression of Vegfr2 correlated with Cd31 levels, suggesting that Kctd10 deficiency promotes pathological angiogenesis while impairing normal angiogenesis. Furthermore, the upregulation of EMT-associated proteins indicated an enhanced metastatic phenotype and tumor angiogenesis (**Figure 7F**, **Supplementary Figure 11B**). These results suggest that endothelial-specific loss of Kctd10 promotes tumor angiogenesis and metastasis while inhibits normal vascular development.

### The expression of KCTD10 is regulated by m^6^A modification

**Figure 8:**
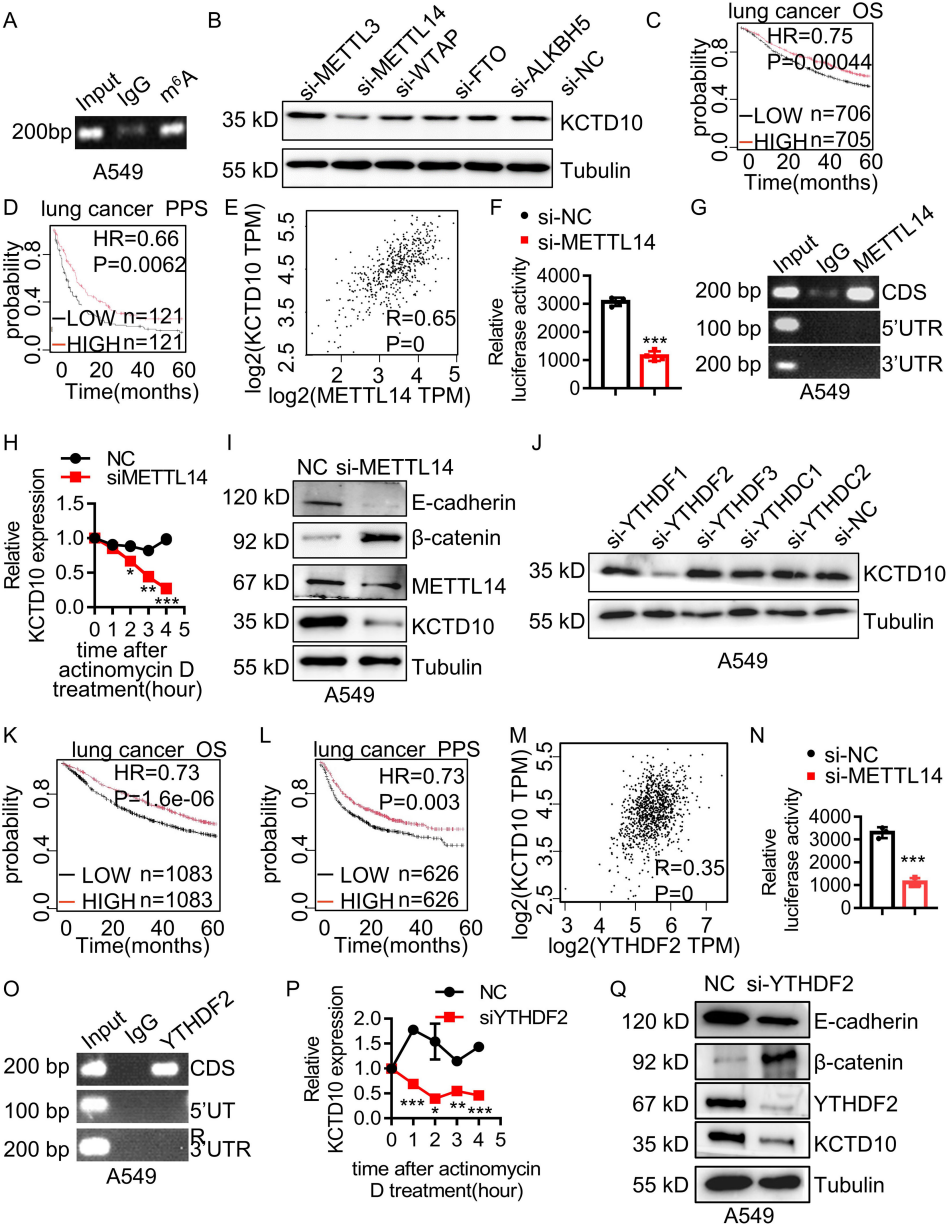
METTL14 and YTHDF2 mediates m^6^A modification of KCTD10 and enhances its mRNA stability. **(A)** MeRIP detecting m^6^A modification of KCTD10 CDS. **(B)** KCTD10 protein levels following m^6^A-related interfering RNAs. **(C, D)** Correlation between METTL14 expression and overall/post-progression survival in lung cancer patients using Kaplan-Meier Plotter survival analysis. **(E)** Correlation between METTL14 and KCTD10 expression in lung cancer using the GEPIA database. **(F)** Effects of METTL14 on the luciferase reporter activity of KCTD10. **(G)** RIP analysis detecting METTL14 binding to the predicted modification site of KCTD10. **(H)** qPCR analysis of KCTD10 RNA stability following METTL14 knockdown. **(I)** Western blot analysis of KCTD10 and related protein expression in A549 cells following METTL14 interference. **(J)** KCTD10 protein expression after m^6^A-related RNA interference. **(K, L)** Correlation between YTHDF2 expression and patient survival. **(M)** The GEPIA database analyzing the correlation between YTHDF2 and KCTD10 expression. **(N)** Effect of YTHDF2 knockdown on luciferase reporter activity of KCTD10. **(O)** RIP analysis of YTHDF2 binding to predicted modification sites of KCTD10 in A549 cells. **(P)** qPCR analysis of KCTD10 RNA stability following YTHDF2 knockdown. **(Q)** Western blot analysis of KCTD10 and downstream gene expression following YTHDF2 knockdown. *P < 0.05, **P < 0.01, ***P < 0.001.

To investigate the upstream regulatory mechanism of KCTD10 expression in lung cancer, we explored the m^6^A modification, the most common form of mRNA modification for the regulation of mRNA stability (57). Previous studies have shown that m^6^A modification plays a tumor-suppressive role in lung cancer (58, 59). Bioinformatic analysis predicted potential m^6^A binding sites within the CDS region of KCTD10 (**Supplementary Figure 12**), which was validated by MeRIP assays (**Figure 8A**, **Supplementary Figure 13A**). Knockdown of m^6^A-related genes in A549 and LLC cells demonstrated that METTL14 knockdown significantly reduced KCTD10 expression (**Figure 8B**, **Supplementary Figure 13B**). The GEPIA database revealed lower METTL14 expression in lung cancer tissues (**Supplementary Figure 13C**), and the Kaplan-Meier plotter analysis indicated that high METTL14 expression was associated with improved prognosis (**Figures 8C, D**). A positive correlation between KCTD10 and METTL14 expression was also observed (**Figure 8E**). Luciferase reporter assays confirmed that knockdown of METTL14 specifically decreased KCTD10 CDS reporter activity, but not other regions (**Figure 8F**, **Supplementary Figure 13D**). Next, RIP experiments showed that METTL14 can enrich RNAs corresponding to the KCTD10 CDS region (**Figure 8G**, **Supplementary Figure 13E**). Transcription inhibitor actinomycin D treatment revealed that METTL14 knockdown reduced KCTD10 mRNA stability (**Figure 8H**, **Supplementary Figure 13F**). Furthermore, Western blots showed that knockdown of METTL14 decreased KCTD10 and E-cadherin levels while increased β-catenin expression (**Figure 8I**, **Supplementary Figures 13G, H**), suggesting that METTL14 regulates the KCTD10/β-catenin axis.

We then knocked down m^6^A-associated readers and found that YTHDF2 interference reduced KCTD10 expression (**Figure 8J**, **Supplementary Figure 14A**). Although YTHDF2 expression was not significantly different between lung tumors and non-tumors (**Supplementary Figure 14B**), high YTHDF2 levels were associated with improved survival (**Figures 8K, L**) and positively correlated with KCTD10 expression (**Figure 8M**). Luciferase reporter assays showed knockdown of YTHDF2 reduced the KCTD10 reporter activity (**Figure 8N**, **Supplementary Figure 14C**). RIP experiments demonstrated that YTHDF2 enriches the KCTD10 CDS regions (**Figure 8O**), and actinomycin D treatment indicated that silencing YTHDF2 decreased KCTD10 mRNA stability (**Figure 8P**). Western blots showed that knockdown of YTHDF2 reduced KCTD10 and E-cadherin levels while increased β-catenin expression (**Figure 8Q**, **Supplementary Figures 14D, E**). These results suggest that the METTL14-YTHDF2 axis stabilizes KCTD10 mRNA via m^6^A modification, contributing to KCTD10 downregulation in lung cancer.

## Discussion

KCTD10 has been reported to play different roles in several tumors (32–34), but its mechanism in lung cancer remains unclear. In this study, we identified low KCTD10 expression in lung cancer from the GEPIA database. IHC analysis further confirmed that KCTD10 expression negatively correlates with the pathologic stage of lung cancer. High KCTD10 expression was associated with prolonged overall and post-progression survival in lung cancer patients, indicating the potential clinical significance of KCTD10 in lung cancer diagnosis, treatment and prognosis.

Lung cancer metastasis, particularly to the brain, is a major therapeutic challenge (60–62). EMT is a critical process in tumor progression and metastasis, characterized by reduced tumor cell viscosity and increased their motility and migration (31). During the EMT process, E-cadherin expression was downregulated while the expression of the stromal markers such as Vimentin, N-cadherin and β-catenin was increased (31, 32). Our findings demonstrated that overexpression of KCTD10 inhibits lung cancer cell growth, migration and invasion, suppresses subcutaneous tumor growth, reduces lung tumor nodule formation, and reverses EMT markers in both lung cancer cells and tissues, suggesting that KCTD10 suppresses lung cancer metastasis by regulating the EMT program. 20% to 65% of lung cancer patients develop brain metastases during the course of the disease, which is significantly higher than in other tumor types (60–62). Our data further revealed that overexpression of Kctd10 effectively reduces the colonization of lung tumors in the brain and prolongs mouse survival. These finding suggest that KCTD10 is a promising therapeutic target for lung cancer metastasis.

Previous studies have shown that KCTD10 expression is induced by IFNγ and IL-6 (27, 37, 63, 64), linking KCTD10 to the inflammatory response that associated with immune system recognition and activation *in vivo*. Through IP-MS, we identified β-catenin as a KCTD10-interacting protein. β-catenin activation promotes EMT-related protein expression and facilitates lung cancer metastasis (20). Mechanistically, KCTD10 promotes K48-linked ubiquitination of β-catenin, leading to its proteasomal degradation and the inhibition of the EMT process. EMT is also associated with upregulation of the immune checkpoint protein PD-L1 (65). PD-L1 is overexpressed on the surface of almost all tumor cells (66). PD-L1 could bind to the PD-1 receptor on the surface of T cells, inhibit T cell function, facilitating immune evasion (67). Since β-catenin enhances PD-L1 and suppresses antitumor immunity (25). We found that overexpression of KCTD10 reduces PD-L1 levels. Tumor infiltrating immune cells (TIICs) play a pivotal role in cancer progression, therapeutic response and overall patient prognosis, and distinct intrinsic subtypes exhibiting heterogeneous immune landscape (68). When combined with anti-PD-1 therapy, KCTD10 overexpression significantly inhibited metastatic lung and brain tumor colonization and led to the strongly improvement in CD8a^+^ T cell infiltration. These results suggest that the KCTD10/β-catenin axis counteracts immune evasion, promotes anti-tumor immunity and improves the efficacy of anti-PD-1 therapy. However, the current study did not extensively dissect the role of KCTD10 between immune activation in specific lung cancer subtypes and immune cell subsets, including regulatory T cells (Tregs). In follow-up studies, we will employ single-cell RNA sequencing and flow cytometry to comprehensively profile immune cell composition and activation states, further enabling a deep understanding of how KCTD10 shapes the tumor immune microenvironment and influences immune responses in subtype-specific contexts.

Tumor angiogenesis in the tumor microenvironment, essential for tumor growth and metastasis, requires the formation of new blood vessels (69). Cancer-associated fibroblasts (CAFs) release stromal cell-derived factors and angiogenic factors, and promote tumor cell growth and blood vessel formation. Vascular endothelial cells mainly mediate the regeneration of tumor blood vessels (55). Vascular normalization, characterized by increased pericyte coverage, improves the hypoxic microenvironment and enhances transport efficiency, thereby enhancing the therapeutic efficacy (54). Aberrant β-catenin activation promotes tumor metastasis and angiogenesis (70–73), while β-catenin/TCF/LEF-dependent transcription, activated by the PI3K/AKT pathway, enhances VEGF-induced angiogenesis (74). In the tumor microenvironment, endothelial cells primarily regulate the new tumor angiogenesis (55). Our study revealed that endothelial Kctd10 knockout in mice accelerates lung cancer progression and tumor angiogenesis. Consistently, the global Kctd10 knockout displayed severe defects in mouse embryonic angiogenesis (28, 29). Kctd10 exerts the function of tissue heterogeneity in regulating angiogenesis in both normal lung tissues and the tumor microenvironment, KCTD10 could promote tumor vascular normalization, making it a potential therapeutic target. The effect of endothelial-specific Kctd10 knockout on β-catenin expression likely reflect a non–cell-autonomous effect, in which the loss of Kctd10 in endothelial cells indirectly influences adjacent tumor and stromal cells through changes in the TME. Specifically, endothelial cells actively participate in cell–cell communication through the secretion of paracrine factors such as Wnt ligands, VEGF, and various cytokines (75, 76). Loss of Kctd10 in endothelial cells may disrupt this paracrine balance or compromise vascular integrity, thereby modulating β-catenin signaling in neighboring cells, suggesting the importance of endothelial–tumor cell crosstalk in mediating the systemic effects of endothelial gene perturbations.

The m^6^A methylation plays diverse roles in lung cancer by regulating different target genes (58, 59). The m^6^A modification regulates sphingolipid metabolism after birth, which correlates with KCTD10 expression (77, 78). The m^6^A methyltransferase METTL14 suppresses lung cancer growth and metastasis through downregulating LINC02747 (79), while the m^6^A reader YTHDF2 is associated with better outcome in NSCLC (80). Although YTHDF2 generally facilitates mRNA degradation (81), several studies have shown that YTHDF2 can also stabilize m^6^A-modified mRNA (82–86). We identified an m^6^A-binding site in the KCTD10 CDS region, where METTL14-YTHDF2 enhances KCTD10 mRNA stability. Both METTL14 and YTHDF2 exhibit low expression in lung cancer but are positively correlated with better patient prognosis, suggesting that impaired METTLE14-YTHDF2 activity contributes to the downregulation of KCTD10 in lung cancer.

In conclusion, KCTD10 suppresses lung cancer metastasis and tumor angiogenesis by interacting with β-catenin to promote its ubiquitin-dependent degradation, which then inhibits EMT and PD-L1 expression, leading to the improving outcome of anti-PD-1 therapy. The dual role of KCTD10 in tumor cells and the tumor microenvironment was demonstrated through lung cancer mouse models and conditional Kctd10 knockout studies. The METTL14-YTHDF2 axis enhances KCTD10 mRNA stability via m^6^A modification, clarifying the regulatory mechanisms of low KCTD10 expression in lung cancer. These findings establish KCTD10 as a promising target for inhibiting lung cancer metastasis and enhancing immunotherapy efficacy. Rational drug design aimed at developing specific KCTD10 activators may represent a novel and effective strategy for lung cancer treatment.

## Data Availability

The raw proteomics data have been deposited and can be accessed via IPX0012893000 at https://www.iprox.cn/. Additional data are available in the Supplementary Information.

## Glossary

AKT: AKT serine/threonine kinase 1
α-sma: α-smooth muscle actin
β-catenin: cadherin-associated protein, beta 1
CBP: CREB-binding protein
CD31: platelet and endothelial cell adhesion molecule 1
CD8: CD8 subunit alpha
CDS: coding sequence
CHX: cycloheximide
c-Myc: cellular myelocytomatosis oncogene
co-IP: co-immunoprecipitation
Cre: cyclization recombination enzyme
DAB: diaminobenzidine
DMEM: dulbecco’s modified eagle medium
E-cadherin: epithelial cadherin
EMT: epithelial-mesenchymal transition
EGF: epidermal growth factor
FFPE: formalin fixed paraffin embedded
GEPIA: Gene Expression Profiling Interactive Analysis
GIST: gastrointestinal-stromal-tumor
GO: gene ontology
HCC: hepatocellular carcinoma
HE: hematoxylin-eosin staining
HER2: human epidermal growth factor receptor 2
HR: hazard ratio
IgG: immunoglobulin G
IHC: immunohistochemistry
IP-MS: immunoprecipitation-mass spectrometry
KCTD10: potassiumchanneltetramerisationdomain-containing10
KEGG: Kyoto encyclopedia of genes and genomes
LUAD: lung adenocarcinoma
LUSC: lung squamous cell carcinoma
M2: alternatively activated macrophages
m6A: n6-methyladenosine
Me-rip: methylated RNA immunoprecipitation
METTL14: methyltransferase like 14
MOI: multiplicity of infection
mRNA: messenger ribonucleic acid
NC: negative control
N-cadherin: neural cadherin
OS: overall survival
P53: tumor suppressor protein, oncogene protein
PCNA: proliferating cell nuclear antigen
PCR: polymerase chain reaction
PD-1: programmed cell death protein 1
PDIP1: polymerasedelta-interactingprotein1
PD-L1: programmed cell death-ligand 1
PPS: post progression survival
PVDF: polyvinylidene fluoride
rac1: ras related C3 botulinum toxin substrate 1
RT-qPCR: reverse transcription-quantitative PCR
SDF-1: stromal cell derived factor 1
SDS: sodium dodecyl sulfate
SiRNA: small interfering RNA
SOE-PCR: splicing overlapping extension PCR
SPF: specific pathogen free
SRAMP: sequence-based RNA adenosine methylation site predictor
STAT3: signal transducer and activator of transcription 3
TCF/LEF: transcription factor/lymphoid enhancer-binding factor
VEGF: vascular endothelial growth factor
YTHDF2: YTH domain-containing family protein 2
ZEB1: zinc finger E-box binding homeobox 1.
