# Supplementary material for: KCTD10 inhibits lung cancer metastasis and angiogenesis via ubiquitin-mediated β-catenin degradation
Source: Front Immunol. 2025 Aug 12;16:1630311. doi: 10.3389/fimmu.2025.1630311 (PMC12378768; doi:10.3389/fimmu.2025.1630311)
Supplement: Supplementary file 1 [file Table1.docx]

Supplementary Materials for **KCTD10 inhibits lung cancer metastasis and angiogenesis via ubiquitin-mediated β-catenin degradation**

Zihao Yin^1,2^, Shengwen Long^1,2^, Hao Zhou^1,2^, Mi Ouyang^1,2^, Qinghao Wang^1,2^, Jun He^3^, Rongyu Su^1^, Zhiwei Li^1,2^, Xiaofeng Ding^1,4,5†^, Shuanglin Xiang^1,2†^

**Affiliations:**

1 The National & Local Joint Engineering Laboratory of Animal Peptide Drug Development, College of Life Science, Hunan Normal University, Changsha, 410081, China

2 State Key Laboratory of Developmental Biology of Freshwater Fish, College of Life Science, Hunan Normal University, Changsha, 410081, China

3 Hunan Provincial Key Laboratory of Regional Hereditary Birth Defects Prevention and Control, Changsha Hospital for Maternal & Child Health Care Affiliated to Hunan Normal University, Changsha, 410007, China

4 Institute of Interdisciplinary Studies, Hunan Normal University, Changsha, 410081, China

5 Peptide and small molecule drug R&D platform, Furong Laboratory, Hunan Normal University, Changsha,410081, China.

† **Correspondence**: Xiaofeng Ding, Ph. D., dingxiaofeng@hunnu.edu.cn, Shuanglin Xiang, Ph. D., xshlin@hunnu.edu.cn

**This supplementary file has 15 figures and 6 tables.**

**Supplementary Figures and Figure Legends**


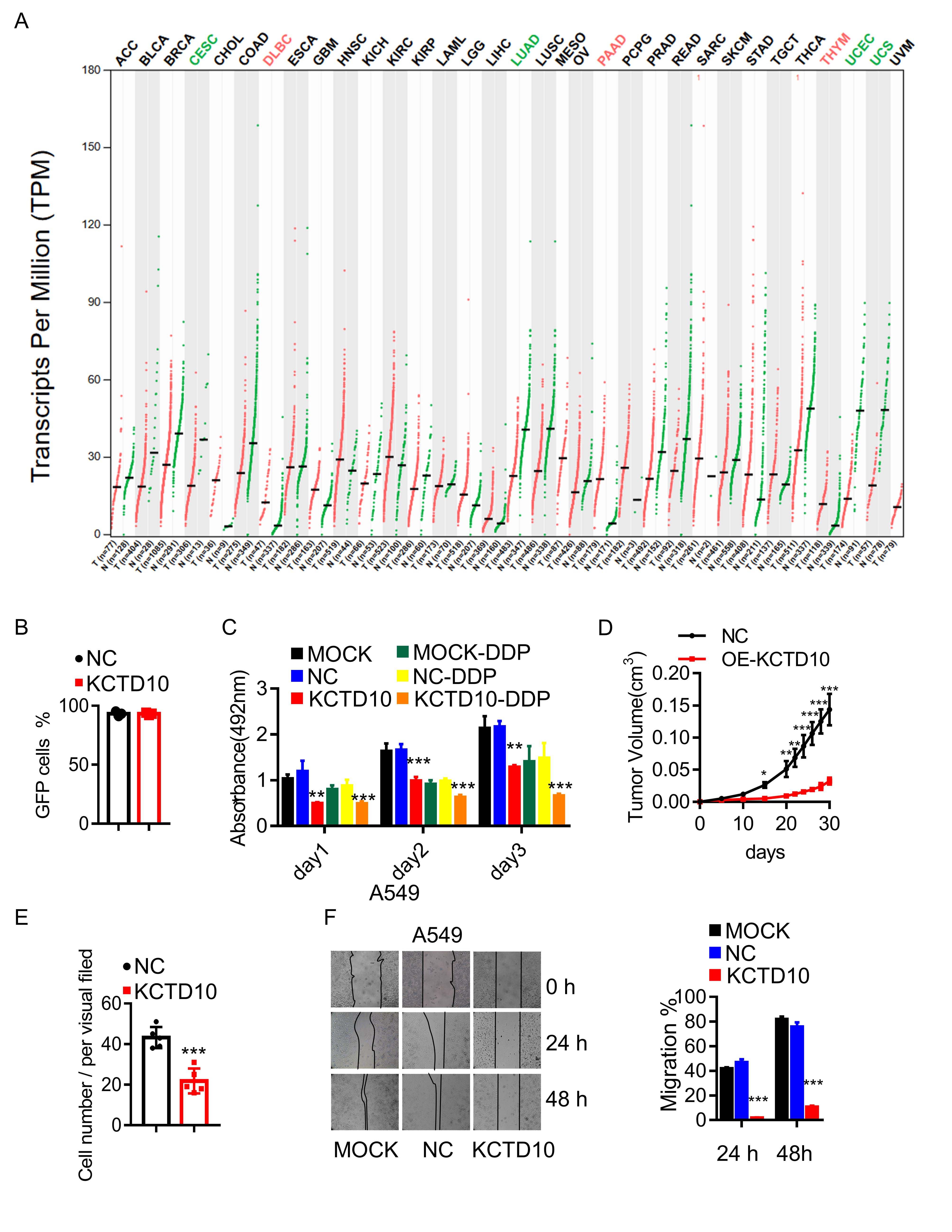


**Fig. S1** Overexpression of KCTD10 inhibits the growth and migration capacity of A549 cells, and enhances the therapeutic effect of cisplatin. **(A)** Pan-cancer analysis of KCTD10 expression from TCGA data base. Tumor types marked in green and red represent statistically significant differences in expression. **(B)** Quantification of GFP fluorescence intensity from Figure 2A. **(C)** MTT assay showing the effect of KCTD10 overexpression on cisplatin sensitivity in A549 cells. **(D)** Growth curve of subcutaneous tumors in nude mice. Mice tumor volume was measured every five days within the first 20 days post-injection and every two days thereafter. Volume was calculated using the formula V = π/6 × L (length) × W (width) × H (height). **(E)** Quantification of the tumor growth curve shown in Figure 2F. **(F)** Wound-healing assay demonstrating the effect of KCTD10 overexpression on A549 cell migration.


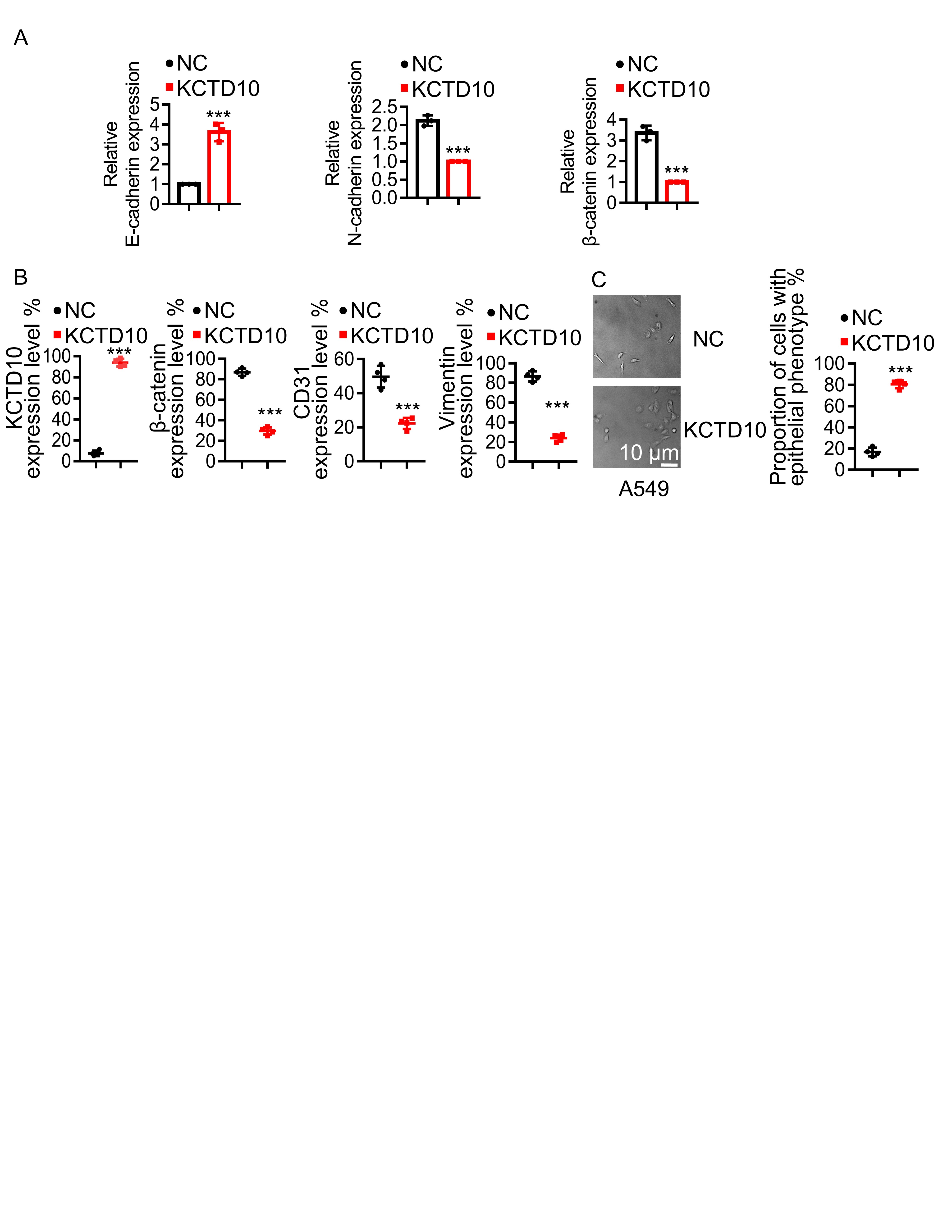


**Fig. S2** Overexpression of KCTD10 affected the expression of EMT-related proteins and CD31. **(A)** Quantification of EMT marker expression by Western blot, corresponding to Figure 2K. **(B)** Quantification of IHC staining, corresponding to Figure 2L. **(C)** Representative images showing morphological changes in A549 cells following KCTD10 overexpression.


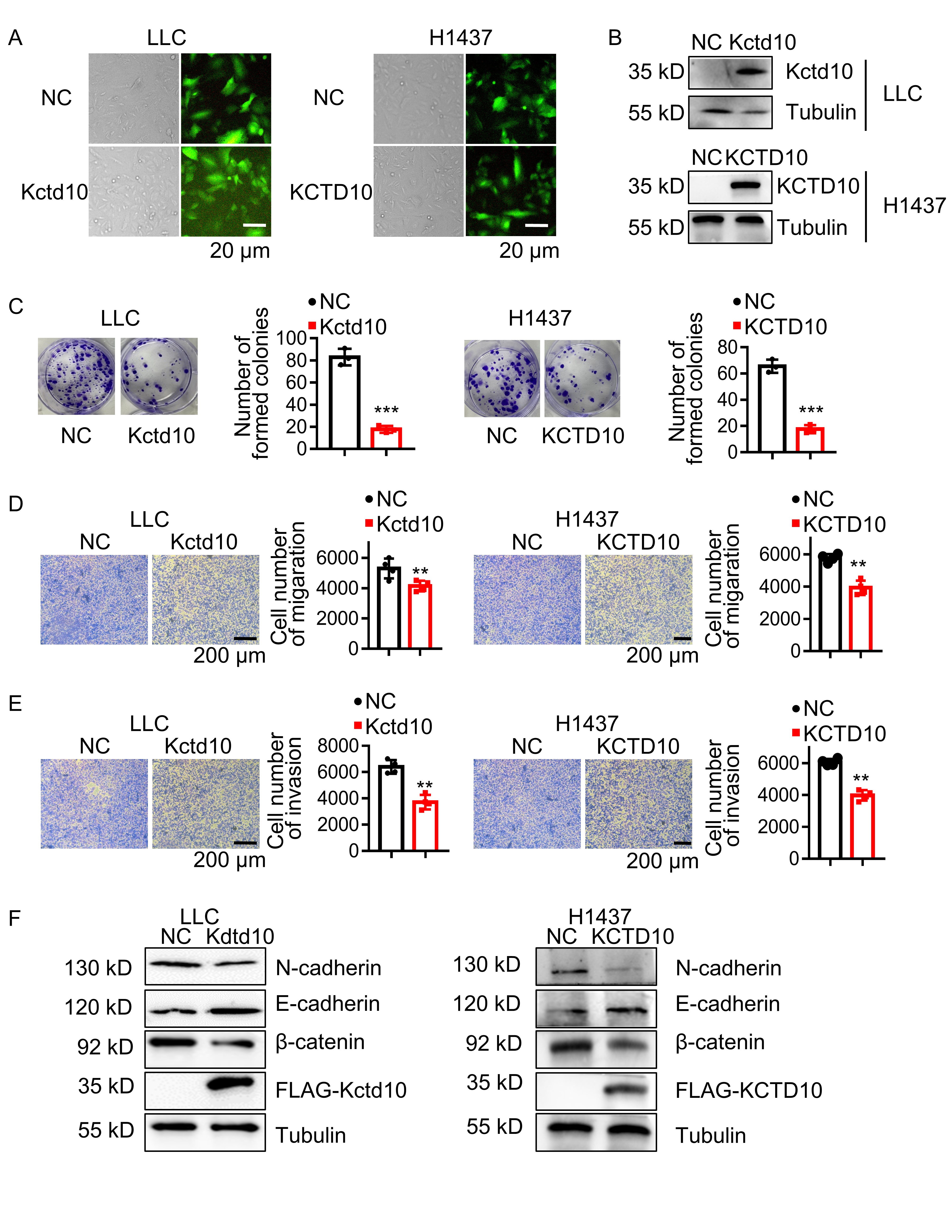


**Fig. S3** Overexpression of Kctd10 inhibits the growth, migration and invasion of LLC and H1437 cells. **(A)** Fluorescence image showing the overexpression of Kctd10 in LLC and H1437 cells. **(B)** Western blots confirming the overexpression of Kctd10 proteins in LLC and H1437 cells by. **(C)** Effect of Kctd10 overexpression on cell growth detected by colony formation assay. **(D-E)** Transwell assays demonstrating the effect of Kctd10 overexpression on the migration and invasion of LLC and H1437 cells. **(F)** Western blots detecting metastasis-related protein expression.


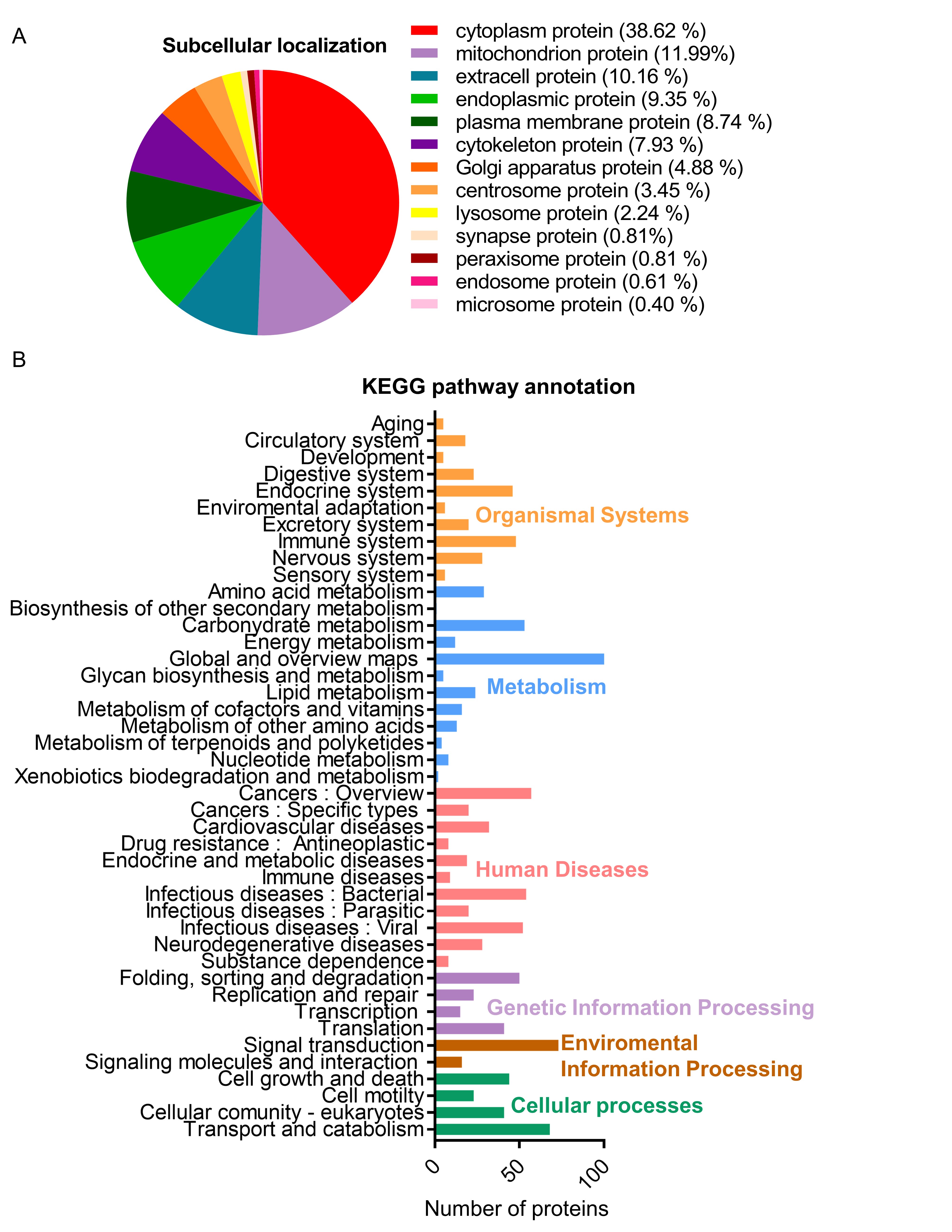


**Fig. S4** Mass-spectrometry analysis of Nucleocytoplasmic distribution of KCTD10-interacting proteins and KEGG pathway annotation. **(A)** Nucleocytoplasmic distribution of the KCTD10-interacting proteins. **(B)** KEGG pathway annotation of KCTD10-interacting proteins.


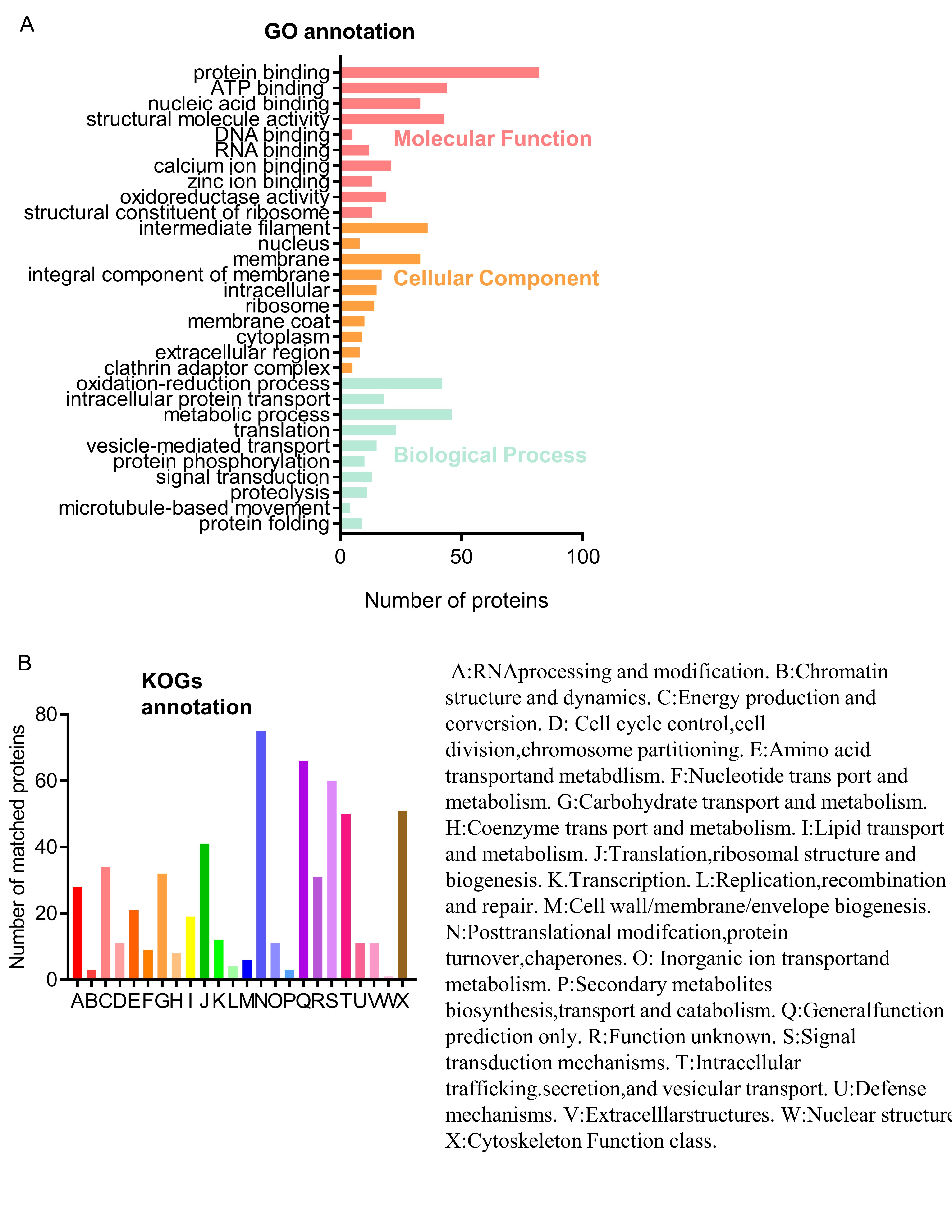


**Fig. S5** Mass spectrometry analysis of GO annotation, KOGs function annotation, IPR annotation and Transfactor prediction. **(A)** GO annotation of KCTD10-interacting proteins, including biological process, cellular component and molecular function. **(B)** KOGs function classification of KCTD10-interacting proteins.

**
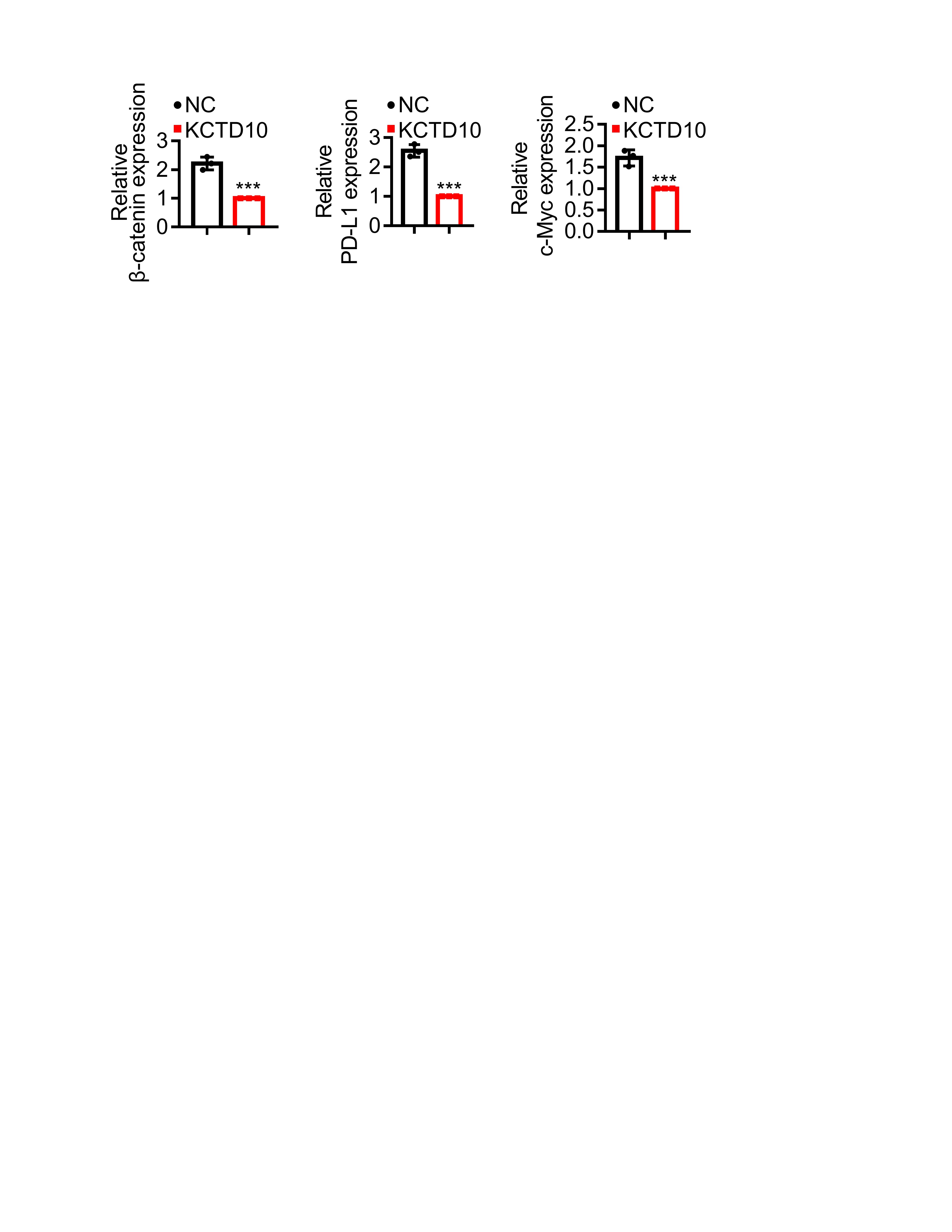
**

**Fig. S6 Overexpression of KCTD10 inhibits the expression of β-catenin and PD-L1. Statistical analysis of Western blot results corresponding to Figure 3L.**

**
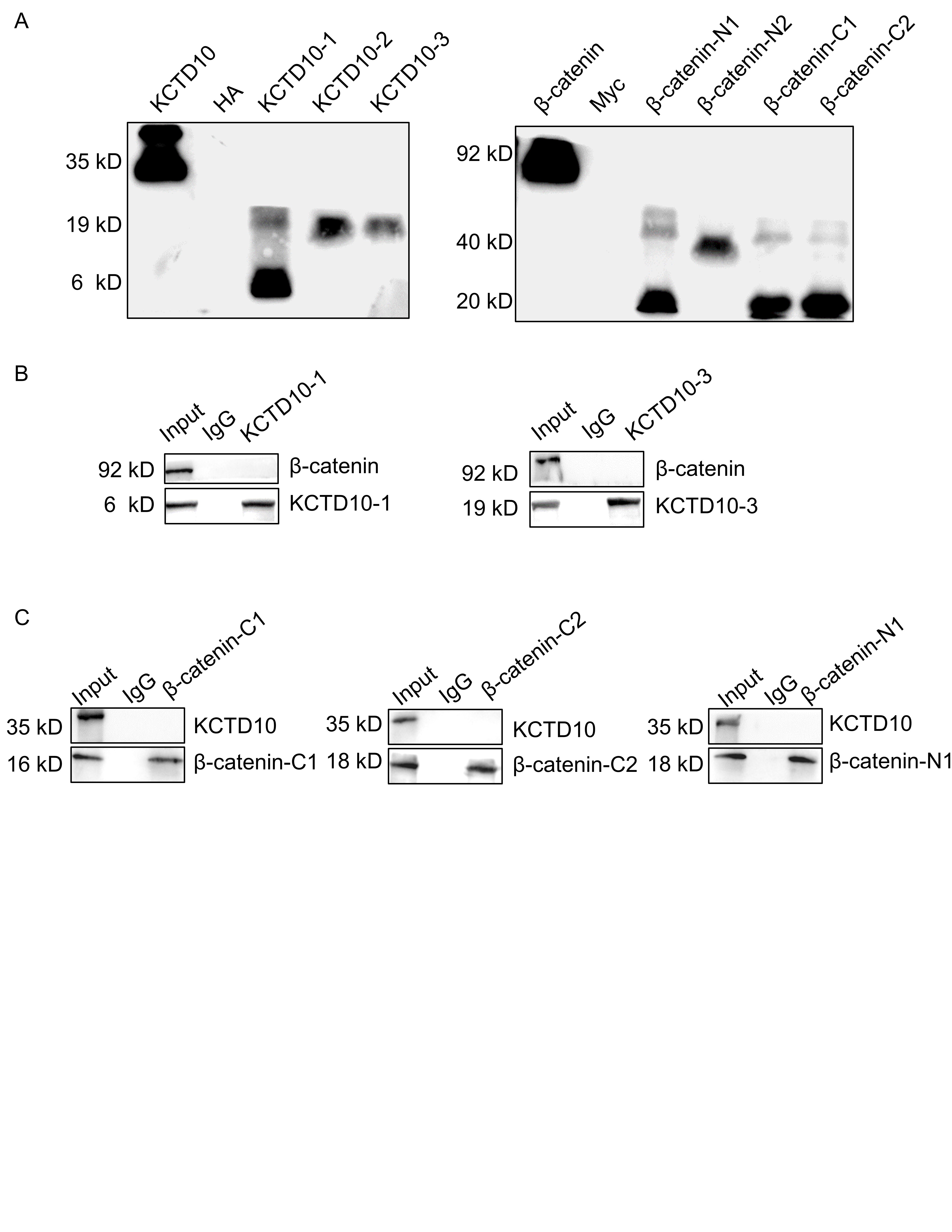
**

**Fig. S7** Immunoprecipitation analysis of KCTD10 and β-catenin binding regions. **(A)** Expression of full-length and truncated fragments of KCTD10 and β-catenin. **(B)** Interaction between truncated KCTD10 proteins and β-catenin proteins. **(C)** Interaction between KCTD10 proteins and truncated β-catenin proteins.


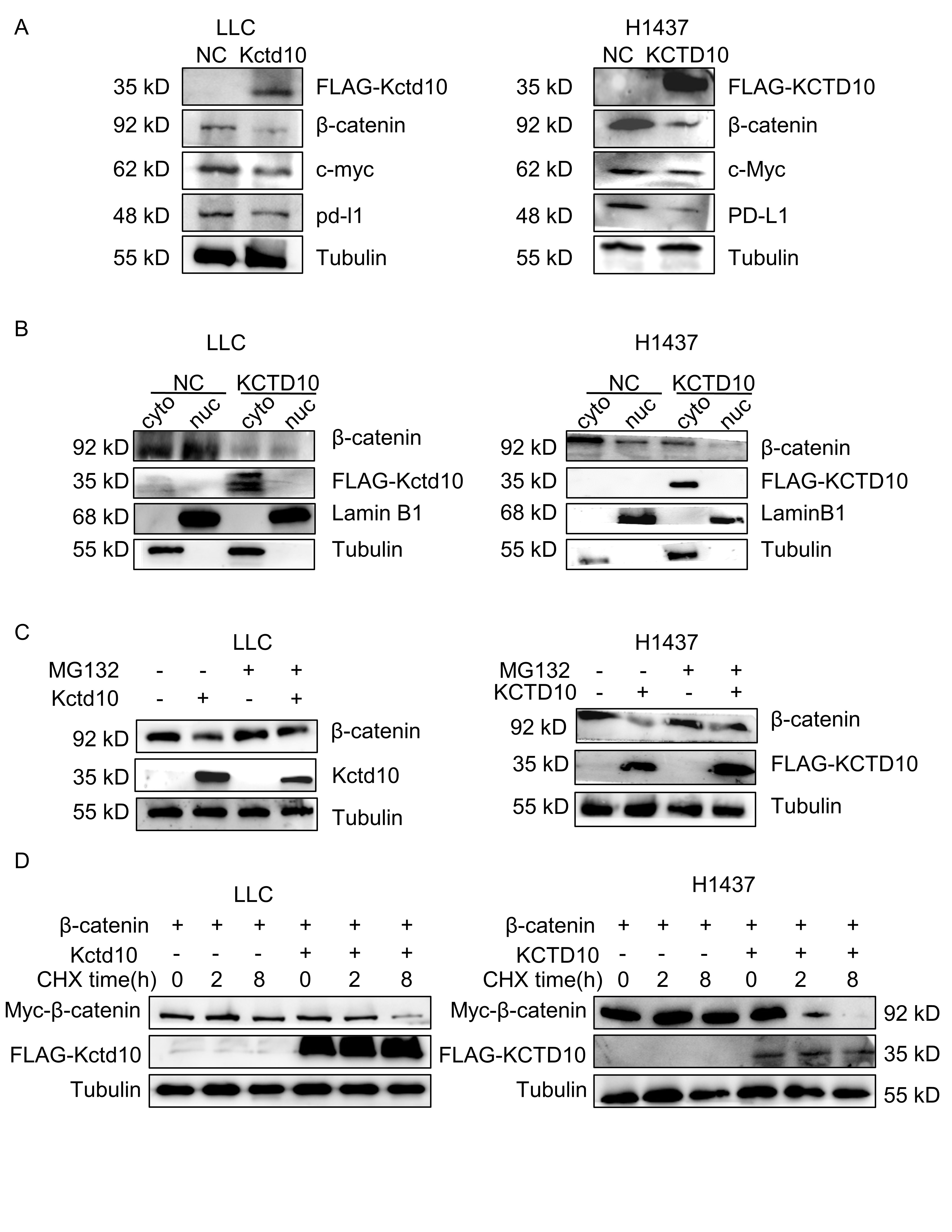


**Fig. S8** Overexpression of Kctd10 promotes the degradation of β-catenin in LLC and H1437 cells. **(A)** Western blot analysis of β-catenin and its downstream protein. **(B)** Effects of KCTD10 overexpression on nucleoplasmic distribution and β-catenin expression measured by nucleocytoplasmic fractionation assay and Western blot. **(C)** Expression of β-catenin following Kctd10 overexpression and MG132 treatment. **(D)** Degradation of β-catenin following CHX treatment and overexpression of Kctd10.


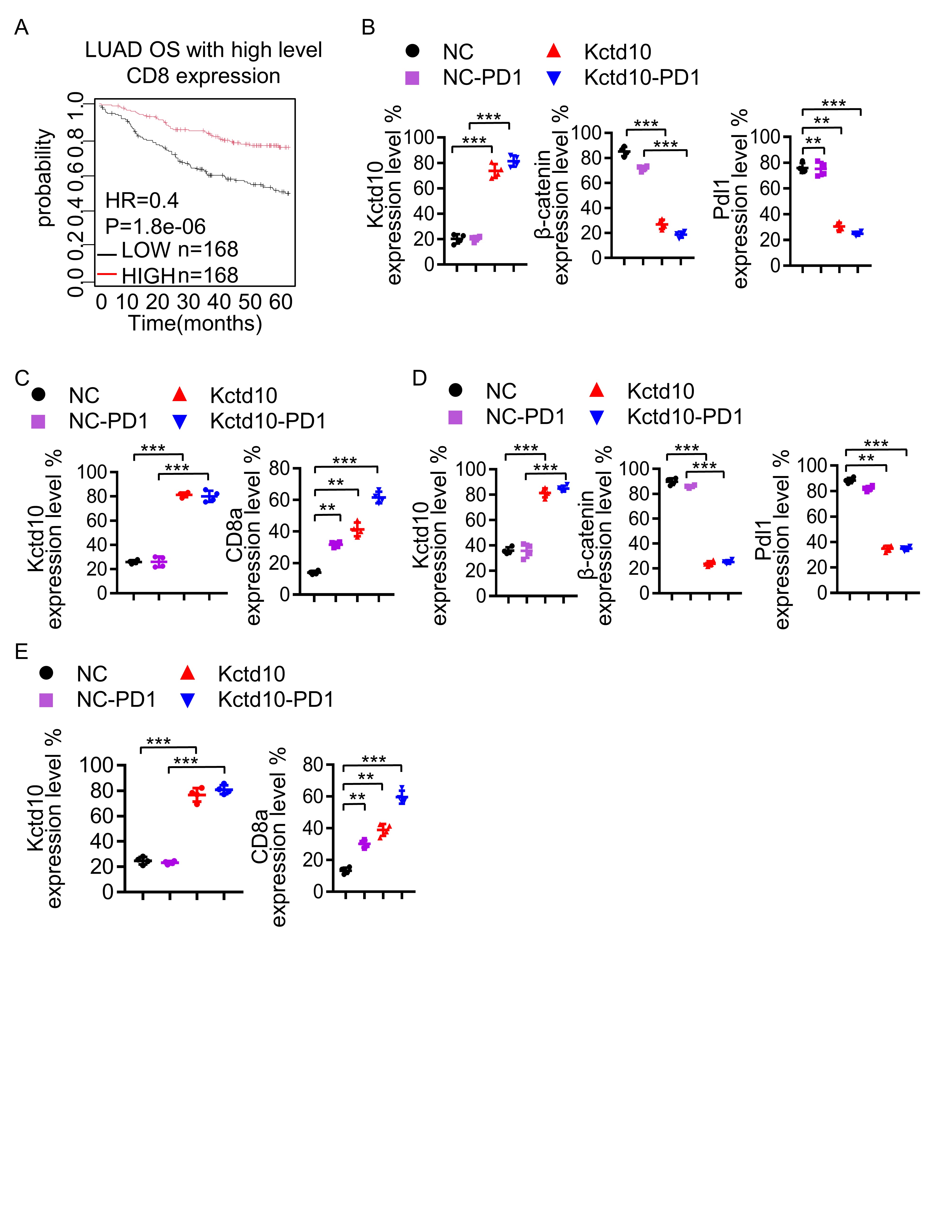


**Fig. S9** Effect of KCTD10 combined anti-PD-1 therapy on CD8a⁺ T cell infiltration. **(A)** Kaplan–Meier survival analysis of LUAD patients with high CD8 expression, stratified by KCTD10 expression levels. **(B)** Quantification of IHC data corresponding to Figure 5G. **(C)** Quantification of CD8a⁺ T cells by IF, corresponding to Figure 5H. **(D)** Statistical analysis of IHC staining shown in Figure 6D. **(E)** Statistical analysis of CD8a IF staining shown in Figure 6E.


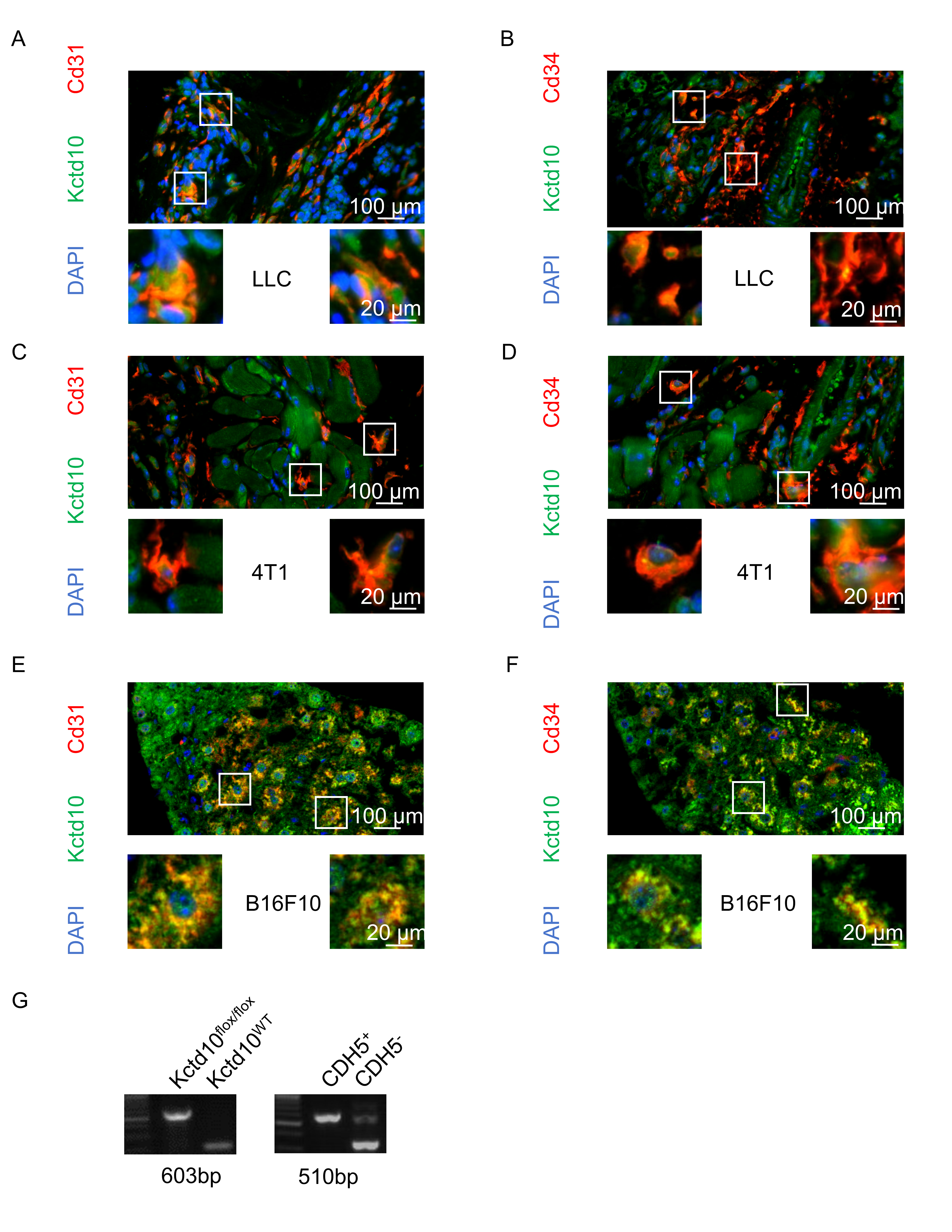


**Fig. S10** Potential link between Kctd10 and tumor angiogenesis. **(A-B)** Colocalization of Kctd10 with Cd31 and Cd34 in subcutaneous tumors of lung cancer cells. **(C-D)** Colocalization of Kctd10 with Cd31 and Cd34 in subcutaneous tumors of breast cancer cells. **(E-F)** Colocalization of Kctd10 with Cd31 and Cd34 in subcutaneous tumors of melanoma cells. **(G)** Identification strategy of Kctd10^flox/flox^CDH5^CreERT2/+^.


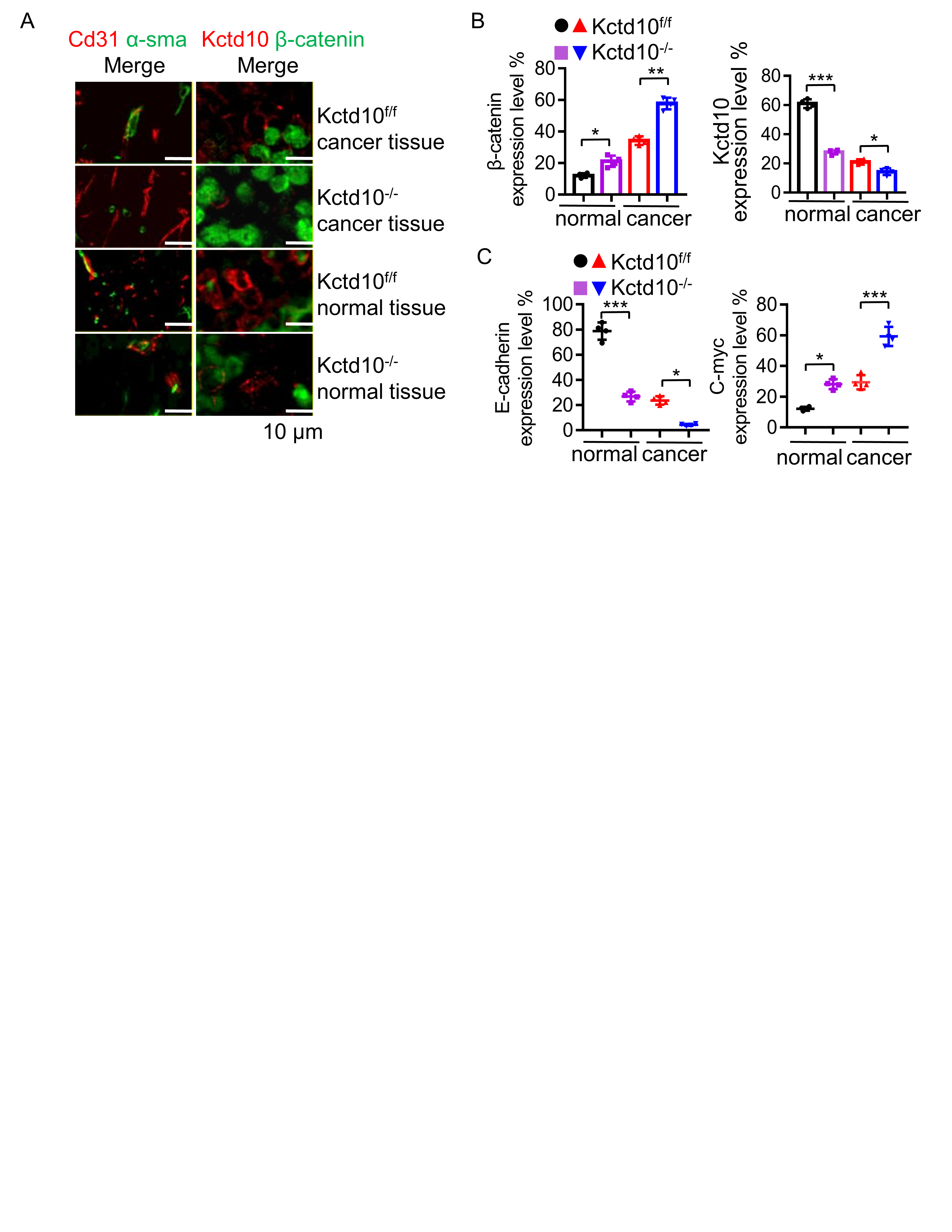


**Fig. S11** The loss of Kctd10 in endothelial cells alters the expression of key target genes in both normal lung tissues and lung tumor tissues. **(A)** Merged IF image corresponding to Figure 7D. **(B)** Statistical analysis of Figure 7E. **(C)** Quantification of IHC data, corresponding to Figure 7F.

**
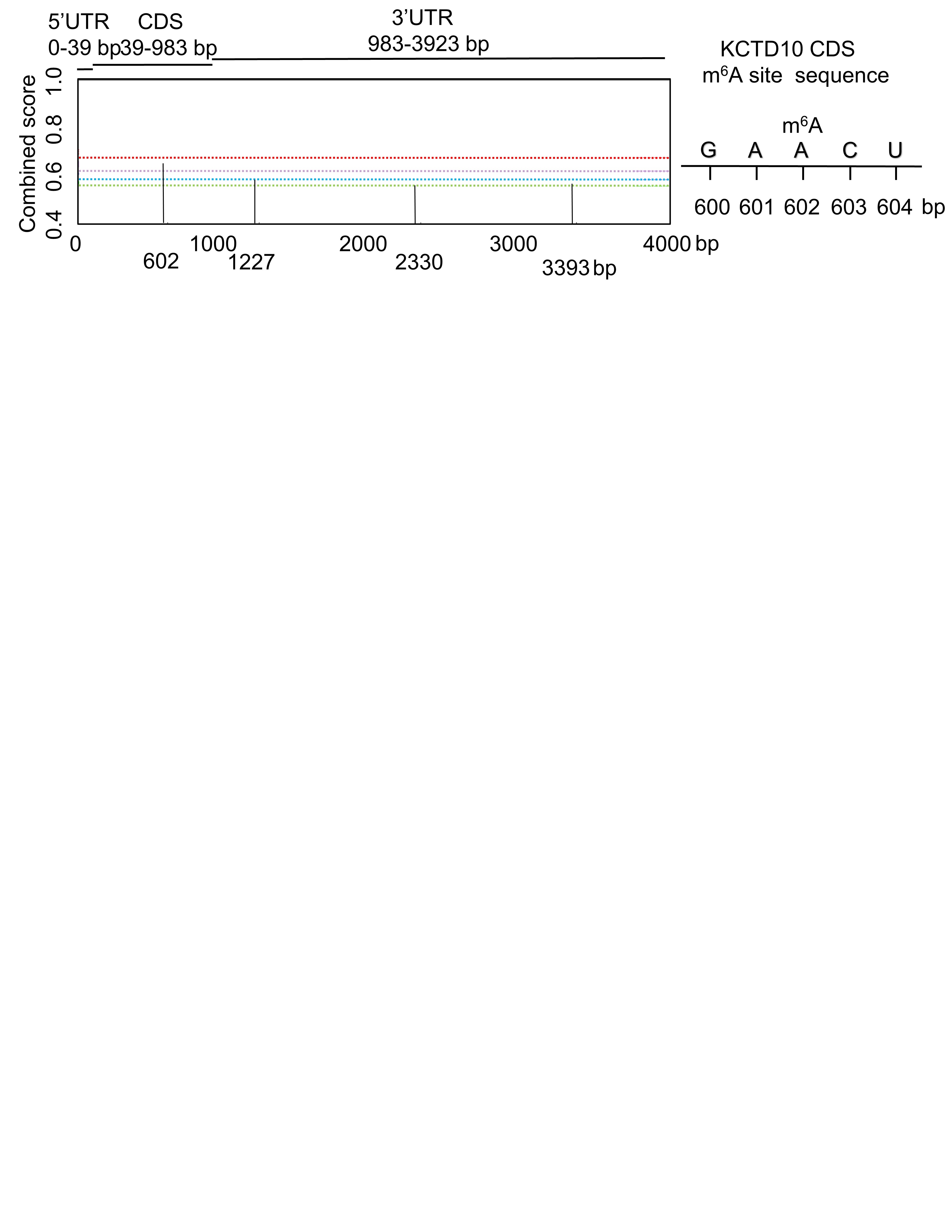
**

**Fig. S12** Predicted m6A modification sites of KCTD10. Predicted m6A binding sites and structural features of KCTD10 mRNA identified using the SRAMP online tool. Nucleotide sequence of the predicted m6A site located in the coding sequence (CDS) region of KCTD10.


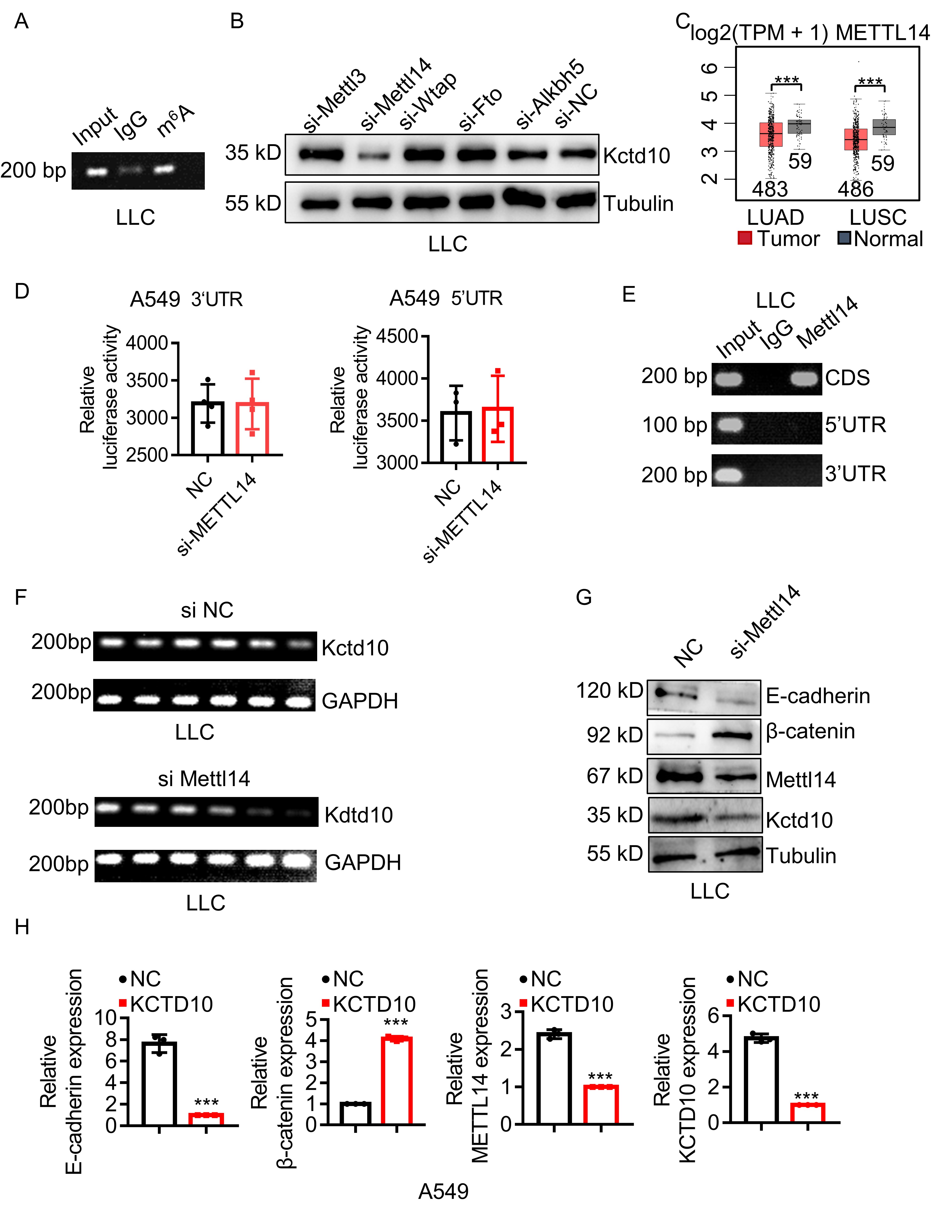


**Fig. S13** METTL14 mediates m^6^A modification of Kctd10 and increases Kctd10 mRNA stability in LLC cells. **(A)** MeRIP analysis of m^6^A modification of Kctd10 CDS. **(B)** Effect of m^6^A-related writer genes on Kctd10 expression. **(C)** Expression levels of METTL14 in lung cancer tissues compared to normal tissues from the GEPIA database. **(D)** Luciferase reporter assays of KCTD10 after METTL14 knockdown. **(E)** RIP assays detecting the binding of Mettl14 to Kctd10. **(F)** Semiquantitative PCR results showing the effect of Mettl14 knockdown on Kctd10 mRNA expression. **(G)** Effect of Mettl14 knockdown on the Kctd10-β-catenin signaling axis detected by western blots. **(H)** Quantification of Western blot results corresponding to Figure 8I.

**
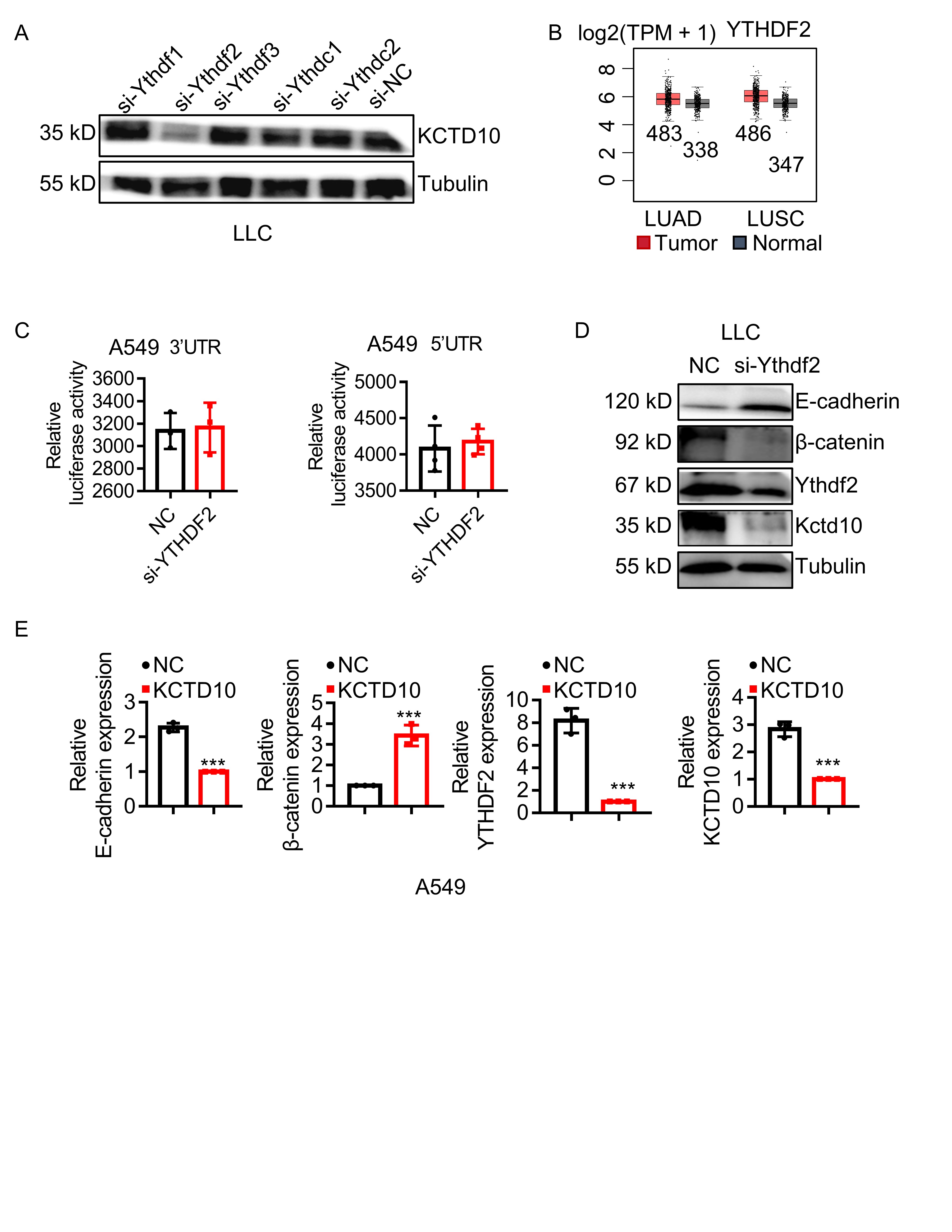
**

**Fig. S14** YTHDF2 mediates m^6^A modification of Kctd10 and increases Kctd10 mRNA stability in LLC cells. **(A)** Effect of m^6^A-related reader gene on Kctd10 expression. **(B)** Ythdf2 expression levels in lung cancer compared to normal tissues from the GEPIA database. **(C)** Luciferase reporter assays of Kctd10 after METTL14 knockdown. **(D)** Effect of Ythdf2 knockdown on the Kctd10-β-catenin signaling axis. **(E)** Quantification of Western blot results corresponding to Figure 8Q.


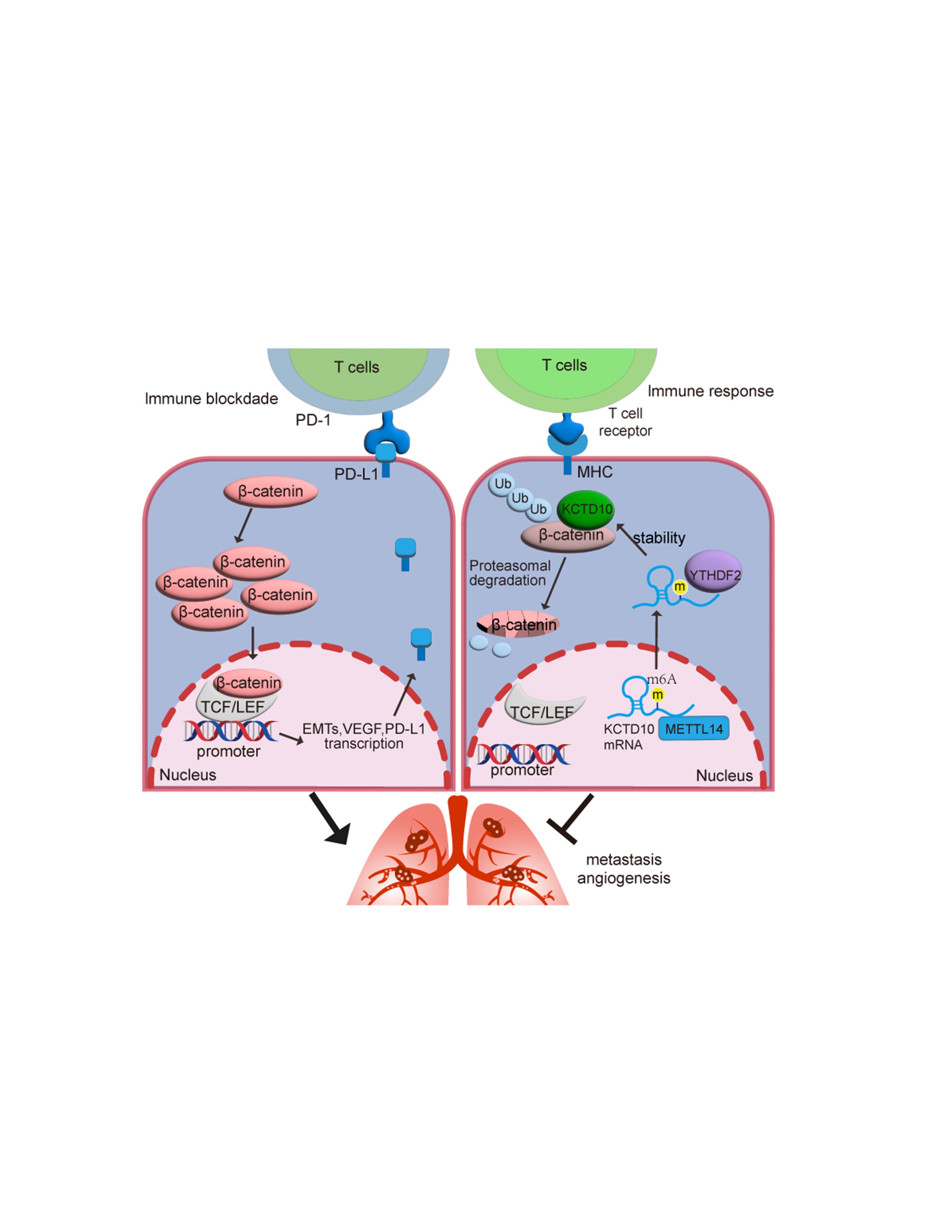


**Fig. S15** Schematic representation of KCTD10 regulation in lung cancer. When KCTD10 expression is low, β-catenin accumulates in the cytoplasm, enters the nucleus, activates the transcription of its downstream TCF/LEF, leading to the upregulation of EMT-related proteins and VEGF, promoting tumor metastasis and angiogenesis. β-catenin binds to the PD-L1 promoter, enhancing its transcription and suppressing the anti-tumor immune response. Conversely, in the presence of high KCTD10 expression, KCTD10 binds to β-catenin in the cytoplasm and promotes its ubiquitination-mediated degradation, preventing its nuclear accumulation, thereby inhibiting tumor metastasis and angiogenesis while enhancing anti-tumor immunity activity. METTL14 enhances KCTD10 expression via m6A modification, stabilizing KCTD10 mRNA in a YTHDF2-dependent manner.

**Table S1. Antibodies used in this study.**

| **Antibody** | **Catalog number** | **Company** | **Purpose** |
| --- | --- | --- | --- |
| KCTD10 | 27279-1-AP | Proteintech | WB |
| KCTD10 | HPA014273 | Sigma | IHC,IF |
| β-catenin | ET1601-5 | HUABIO | WB,IHC,IP,IF |
| β-catenin | EM0306 | HUABIO | IP |
| CD8α | 70306 | Cell Signaling | IHC |
| PD-L1 | ab228415 | Abcam | WB,IHC |
| IgG | ab37355 | Abcam | IP |
| METTL14 | ab309096 | Abcam | WB,IP |
| CD31 | ab9498 | Abcam | IHC,IF |
| Tubulin | AF7010 | Affinity Biosciences | WB |
| c-Myc | 380784 | Zenbio | WB,IHC |
| Cyclin-D1 | R380999 | Zenbio | WB |
| Vimentin | 240140 | Zenbio | IHC |
| α-sma | 250104 | Zenbio | IF |
| VEGFR2 | A5609 | ABclonal | IHC |
| E-cadherin | A3044 | ABclonal | WB,IHC |
| N-cadherin | A0433 | ABclonal | IHC |
| Ubiquitin | sc-8017 | Santa Cruz Biotech | WB |
| Myc-tag | C3956 | Sigma | IP,WB |
| Flag-tag | F2555 | Sigma | WB |
| HA-tag | [05-904](https://www.sigmaaldrich.cn/CN/zh/product/mm/05904) | Sigma | IP,WB |
| M^6^A | A19841 | ABclonal | me-RIP |

**Table S2**. **KCTD10 expression and clinical characteristics**.

| Clinical features | Number | KCTD10 | | | P Value |
| --- | --- | --- | --- | --- | --- |
|  |  | Overexpression | Low expression | No expression |  |
| Total number | 80 | 16 | 14 | 30 |  |
| Gender |  |  |  |  | 0.9163 |
| Female | 17 | 11 | 3 | 3 |  |
| Male | 63 | 41 | 16 | 6 |  |
| Age |  |  |  |  | 0.8446 |
| (median, 55.3 years) |  |  |  |  |  |
| ﹤ | 37 | 28 | 5 | 4 |  |
| ≥ | 43 | 24 | 14 | 5 |  |
| Histological diagnosis |  |  |  |  | <0.0001 |
| LUAD | 30 | 17 | 7 | 6 |  |
| LUSC | 22 | 15 | 5 | 2 |  |
| Small cell carcinoma | 8 | 3 | 4 | 1 |  |
| Atypical carcinoid | 2 | 1 | 1 | 0 |  |
| Large cell carcinoma | 4 | 3 | 1 | 0 |  |
| Bronchioloalveolar carcinoma | 2 | 1 | 1 | 0 |  |
| Lung cancer histological Grade |  |  |  |  | <0.0001 |
| Grade I | 37 | 32 | 5 | 0 |  |
| Grade II | 22 | 7 | 10 | 5 |  |
| Grade III | 9 | 1 | 4 | 4 |  |
| Normal lung tissue | 12 | 12 | 0 | 0 |  |

**Table S3. β-catenin expression and clinical characteristics**.

| Clinical features | Number | β-catenin | | | P Value |
| --- | --- | --- | --- | --- | --- |
|  |  | Overexpression | Low expression | No expression |  |
| Total number | 80 | 16 | 14 | 30 |  |
| Gender |  |  |  |  | 0.8964 |
| Female | 17 | 7 | 8 | 2 |  |
| Male | 63 | 35 | 25 | 3 |  |
| Age |  |  |  |  | 0.7954 |
| (median, 55.3 years) |  |  |  |  |  |
| ﹤ | 37 | 23 | 12 | 2 |  |
| ≥ | 43 | 20 | 21 | 3 |  |
| Histological diagnosis |  |  |  |  | <0.0001 |
| LUAD | 30 | 21 | 9 | 0 |  |
| LUSC | 22 | 9 | 11 | 2 |  |
| Small cell carcinoma | 8 | 5 | 2 | 1 |  |
| Atypical carcinoid | 2 | 1 | 1 | 0 |  |
| Large cell carcinoma | 4 | 2 | 2 | 0 |  |
| Bronchioloalveolar carcinoma | 2 | 1 | 1 | 0 |  |
| Lung cancer histological Grade |  |  |  |  | <0.0001 |
| Grade I | 37 | 11 | 24 | 0 |  |
| Grade II | 22 | 20 | 2 | 0 |  |
| Grade III | 9 | 9 | 0 | 0 |  |
| Normal lung tissue | 12 | 2 | 7 | 3 |  |

**Table S4. Primers used in the study.**

| Primers name | Sequences (5’-3’) | Purpose | Fragment size | Plasmid  Vectors |
| --- | --- | --- | --- | --- |
| KCTD10 F | ATGGAAGAGATGTCAGGAGAA | construct full length KCTD10 | 945 bp | pCMV-HA  pCMV-Myc |
| KCTD10 R | TCACTGGTGGAGGTGGGCCCG |  |  |  |
| KCTD10 3′ UTR F | GCAGGCAAGAGACCGAGCCGCCCT | construct KCTD10 3′ UTR | 2940 bp | pGL3-basic |
| KCTD10 3′ UTR R | AGCATTTTATGACTTTTATTTTA |  |  |  |
| β-catenin-N1 F | ATGGCTACTCAAGCTGATTT | construct β-catenin-N1 | 420 bp | pCMV-Myc |
| β-catenin-N1 R | AATCAAGTTTACAACTGCAT |  |  |  |
| β-catenin-N2 F | AACTATCAAGATGATGCAGAA | construct β-catenin-N2 | 1140 bp | pCMV-Myc |
| β-catenin-N2 R | AAGGGCAAGATTTCGAATCA |  |  |  |
| β-catenin-C1 F | TGTCCCGCAAATCATGCACC | construct β-catenin-C1 | 438 bp | pCMV-Myc |
| β-catenin-C1 R | CTCAGACATTCGGAACAAAA |  |  |  |
| β-catenin-C2 F | GACAAGCCACAAGATTACAA | construct β-catenin-C2 | 454 bp | pCMV-Myc |
| β-catenin-C2 R | TTACAGGTCAGTATCAAACCAGGC |  |  |  |
| KCTD10-1 F | ATGGAAGAGATGTCAGGAGAA | construct KCTD10-1 | 96 bp | pCMV-Myc |
| KCTD10-1 R | TTTGGAGCTGGGGCTCGTGCC |  |  |  |
| KCTD10-2 F | ATGGAAGAGATGTCAGGAGAA | construct KCTD10-2 | 399 bp | pCMV-Myc |
| KCTD10-2 R | ATCTTTGTTTTGTAGGGCCGC |  |  |  |
| KCTD10-3 F | ACTTATGAGCCTTTCTGCAAG | construct KCTD10-3 | 546 bp | pCMV-Myc |
| KCTD10-3 R | TCACTGGTGGAGGTGGGCCCG |  |  |  |
| KCTD10 P3 | CGGGAGTGTAGGAACTAGGCTGAA | genotyping | 603 bp |  |
| KCTD10 P4 | CAGGAGCGGAAGATAACACCAAA |  |  |  |
| CDH5CreERT2 F | TTCCCGCAGAACTGAAGATG | genotyping | 510 bp |  |
| CDH5CreERT2 R | CTACACCAGAGACGGAAATCCATC |  |  |  |
| pCDNA3.1-(HA-Ub) F | ATGCAGATCTTCGTGAAAACC | construct WT Ub and full-length mutant Ub | 231 bp | pCDNA3.1 |
| pCDNA3.1-(HA-Ub) R | CTACCACCTCTCAGACGCAGG |  |  |  |
| K6R F | ATGCAGATCTTCGTGAGAACC | construct K6R  mutant Ub | 231 bp | pCDNA3.1 |
| K6R R | CTACCACCTCTCAGACGCAGG |  |  |  |
| K11R F | CCTTACCGGCAGGACC  ATCA | construct K11R  mutant Ub | 177 bp | pCDNA3.1 |
| K11R R | GTCGACTCCTTCTGGA  TGTT |  |  |  |
| K27R Fm | ATGTGAGGGCCAAGATCCAGG | overlapping mutation PCR for K27R | |  |
| K27R Rm | CCTGGATCTTGGCCCTCACAT |  |  |  |
| K33R Fm | GATCCAGGATAGGGAAGGCAT | overlapping mutation PCR for K33R | |  |
| K33R Rm | ATGCCTTCCCTATCCTGGATC |  |  |  |
| K48R Fm | CTTTGCAGGCAGGCAGCTGGA | overlapping mutation PCR for K48R | |  |
| K48R Rm | TCCAGCTGCCTGCCTGCAAAG |  |  |  |
| K63R F | CCTTACCGGCAAGACC  ATCA | construct K63R  mutant Ub | 177 bp | pCDNA3.1 |
| K63R R | GTCGACTCCCTCTGGA  TGTT |  |  |  |
| K6O F | ATGCAGATCTTCGTGAAAACC | construct K6O  mutant Ub | 231 bp | pCDNA3.1 |
| K6O R | CTACCACCTCTCAGACGCAGG |  |  |  |
| K11O F | CCTTACCGGCAAGACC  ATCA | construct K11O  mutant Ub | 177 bp | pCDNA3.1 |
| K11O R | GTCGACTCCTTCTGGA  TGTT |  |  |  |
| K27O Fm | ATGTGAAGGCCAGGATCCAGG | overlapping mutation PCR for K27O | |  |
| K27O Rm | CCTGGATCCTGGCCTTCACAT |  |  |  |
| K33O Fm | GATCCAGGATAAGGAAGGCAT | overlapping mutation PCR for K33O | |  |
| K33O Rm | ATGCCTTCCTTATCCTGGATC |  |  |  |
| K48O Fm | CTTTGCAGGCAAGCAGCTGGA | overlapping mutation PCR for K48O | |  |
| K48O Rm | TCCAGCTGCTTGCCTGCAAAG |  |  |  |
| K63O F | CCTTACCGGCAGGACC  ATCA | construct K63O  mutant Ub | 77 bp | pCDNA3.1 |
| K63O R | GTCGACTCCTTCTGGA  TGTT |  |  |  |
| K0 F | ATGCAGATCTTCGTGAGAACC | construct K0  mutant Ub | 231 bp | pCDNA3.1 |
| K0 R | CTACCACCTCTCAGACGCAGG |  |  |  |
| β-catenin F | ATGGCTACTCAAGCTGATTT | construct full length β-catenin | 2343 bp | pCMV-HA  pCMV-Myc |
| β-catenin R | TTACAGGTCAGTATCAAACCAGGC |  |  |  |
| β-actin F | AAGGAGATCACTGCCCTGGC | RT-PCR | 225 bp |  |
| β-actin R | CCACATCTGCTGGAAGGTGG | RT-PCR |  |  |
| METTL14 F | GAGTGTGTTTACGAAAATGGGGT | RT-PCR | 154 bp |  |
| METTL14 R | CCGTCTGTGCTACGCTTCA | RT-PCR |  |  |
| KCTD10 F | GGGTGGAGCCCTCTACTATAC | RT-PCR | 101 bp |  |
| KCTD10 R | CACTGTCGGTGAGCACTTCC | RT-PCR |  |  |

**Table S5. Animals used in the study.**

| **Animals** | **Company** |
| --- | --- |
| Nude mice (Balb/nu) | Hunan SJA Laboratory Animal Corporation (Changsha, China, http://www.hnsja.com/) |
| C57BL/6J mice | Jackson Laboratories (BarBarbor, ME, https://www.jax.org/) |
| CDH5^CreERT2/+^ C57BL/6J mice | [GemPharmatech](https://cn.gempharmatech.com/about/intro.html#:~:text=GemPharmatech LLC.,GemPharmatech LLC. %EF%BC%88%E4%BB%A5%E4%B8%8B%E7%AE%80%E7%A7%B0%E2%80%9DGemPharmatech%E2%80%9D%EF%BC%89%E6%98%AF%E6%B1%9F%E8%8B%8F%E9%9B%86%E8%90%83%E8%8D%AF%E5%BA%B7%E7%94%9F%E7%89%A9%E7%A7%91%E6%8A%80%E8%82%A1%E4%BB%BD%E6%9C%89%E9%99%90%E5%85%AC%E5%8F%B8%EF%BC%88%E4%B8%8B%E7%A7%B0%E2%80%9C%E9%9B%86%E8%90%83%E8%8D%AF%E5%BA%B7%E2%80%9D%EF%BC%89%E7%9A%84%E5%85%A8%E8%B5%84%E5%AD%90%E5%85%AC%E5%8F%B8%EF%BC%8C%E6%80%BB%E5%8A%9E%E4%BA%8B%E5%A4%84%E4%BD%8D%E4%BA%8E%E7%BE%8E%E5%9B%BD%E5%8A%A0%E5%88%A9%E7%A6%8F%E5%B0%BC%E4%BA%9A%E5%B7%9E%E5%9C%A3%E5%9C%B0%E4%BA%9A%E5%93%A5%E5%B8%82%EF%BC%8C%E4%B8%BB%E8%A6%81%E9%9D%A2%E5%90%91%E7%9A%84%E5%AE%A2%E6%88%B7%E4%B8%BA%E5%9B%BD%E9%99%85%E7%9F%A5%E5%90%8D%E5%A4%A7%E5%9E%8B%E7%94%9F%E7%89%A9%E5%88%B6%E8%8D%AF%E5%85%AC%E5%8F%B8%EF%BC%8CCRO%E6%9C%8D%E5%8A%A1%E6%8F%90%E4%BE%9B%E5%95%86%E3%80%81%E5%A4%A7%E5%AD%A6%E5%92%8C%E7%A0%94%E7%A9%B6%E6%9C%BA%E6%9E%84%E3%80%82) (Nanjing, China, https://cn.gempharmatech.com/) |
| KCTD10^flox/flox^ C57BL/6J mice | [GemPharmatech](https://cn.gempharmatech.com/about/intro.html#:~:text=GemPharmatech LLC.,GemPharmatech LLC. %EF%BC%88%E4%BB%A5%E4%B8%8B%E7%AE%80%E7%A7%B0%E2%80%9DGemPharmatech%E2%80%9D%EF%BC%89%E6%98%AF%E6%B1%9F%E8%8B%8F%E9%9B%86%E8%90%83%E8%8D%AF%E5%BA%B7%E7%94%9F%E7%89%A9%E7%A7%91%E6%8A%80%E8%82%A1%E4%BB%BD%E6%9C%89%E9%99%90%E5%85%AC%E5%8F%B8%EF%BC%88%E4%B8%8B%E7%A7%B0%E2%80%9C%E9%9B%86%E8%90%83%E8%8D%AF%E5%BA%B7%E2%80%9D%EF%BC%89%E7%9A%84%E5%85%A8%E8%B5%84%E5%AD%90%E5%85%AC%E5%8F%B8%EF%BC%8C%E6%80%BB%E5%8A%9E%E4%BA%8B%E5%A4%84%E4%BD%8D%E4%BA%8E%E7%BE%8E%E5%9B%BD%E5%8A%A0%E5%88%A9%E7%A6%8F%E5%B0%BC%E4%BA%9A%E5%B7%9E%E5%9C%A3%E5%9C%B0%E4%BA%9A%E5%93%A5%E5%B8%82%EF%BC%8C%E4%B8%BB%E8%A6%81%E9%9D%A2%E5%90%91%E7%9A%84%E5%AE%A2%E6%88%B7%E4%B8%BA%E5%9B%BD%E9%99%85%E7%9F%A5%E5%90%8D%E5%A4%A7%E5%9E%8B%E7%94%9F%E7%89%A9%E5%88%B6%E8%8D%AF%E5%85%AC%E5%8F%B8%EF%BC%8CCRO%E6%9C%8D%E5%8A%A1%E6%8F%90%E4%BE%9B%E5%95%86%E3%80%81%E5%A4%A7%E5%AD%A6%E5%92%8C%E7%A0%94%E7%A9%B6%E6%9C%BA%E6%9E%84%E3%80%82) (Nanjing, China, https://cn.gempharmatech.com/) |

**Table S6. siRNA sequences in the study.**

| GENE name | Sequences (5’-3’) |
| --- | --- |
| METTL3 | UCGCUUUACCUCAAUCAACUCdTdT |
|  | GUUGAUUGAGGUAAAGCGAGGdTdT |
| ALBKH5 | UCAAAAUAUAUUAGAUUUGGUdTdT |
|  | CAAAUCUAAUAUAUUUUGAAAdTdT |
| WTAP | UUUGAAGUCUGUUUCACUCAAdTdT |
|  | GAGUGAAACAGACUUCAAAGUdTdT |
| METTL14 | AAUCUCUUCUUCAUAUGGCAAdTdT |
|  | GCCAUAUGAAGAAGAGAUUUAdTdT |
| FTO | UAUCGAUUGCCUUGAAACCAAdTdT |
|  | GGUUUCAAGGCAAUCGAUACAdTdT |
| YTHDF1 | UUAUUAUCUUGUCCUUUUGUUdTdT |
|  | UUAUUAUCUUGUCCUUUUGUUdTdT |
| YTHDF2 | UUCCAAUAUCCAUGUUAUGCUdTdT |
|  | CAUAACAUGGAUAUUGGAACUdTdT |
| YTHDF3 | AGAAUAUGGAAAUCCAAUGGAdTdT |
|  | CAUUGGAUUUCCAUAUUCUCUdTdT |
| YTHDC1 | UCAUUUUUAUCUUGUUCACUCdTdT |
|  | GUGAACAAGAUAAAAAUGAGAdTdT |
| YTHDC2 | UCUGAUAACCAAUUGUUUGACdTdT |
|  | CAAACAAUUGGUUAUCAGAUCdTdT |
