# Supplementary figures and images for: KCTD10 inhibits lung cancer metastasis and angiogenesis via ubiquitin-mediated β-catenin degradation
Source: Front Immunol. 2025 Aug 12;16:1630311. doi: 10.3389/fimmu.2025.1630311 (PMC12378768; doi:10.3389/fimmu.2025.1630311)

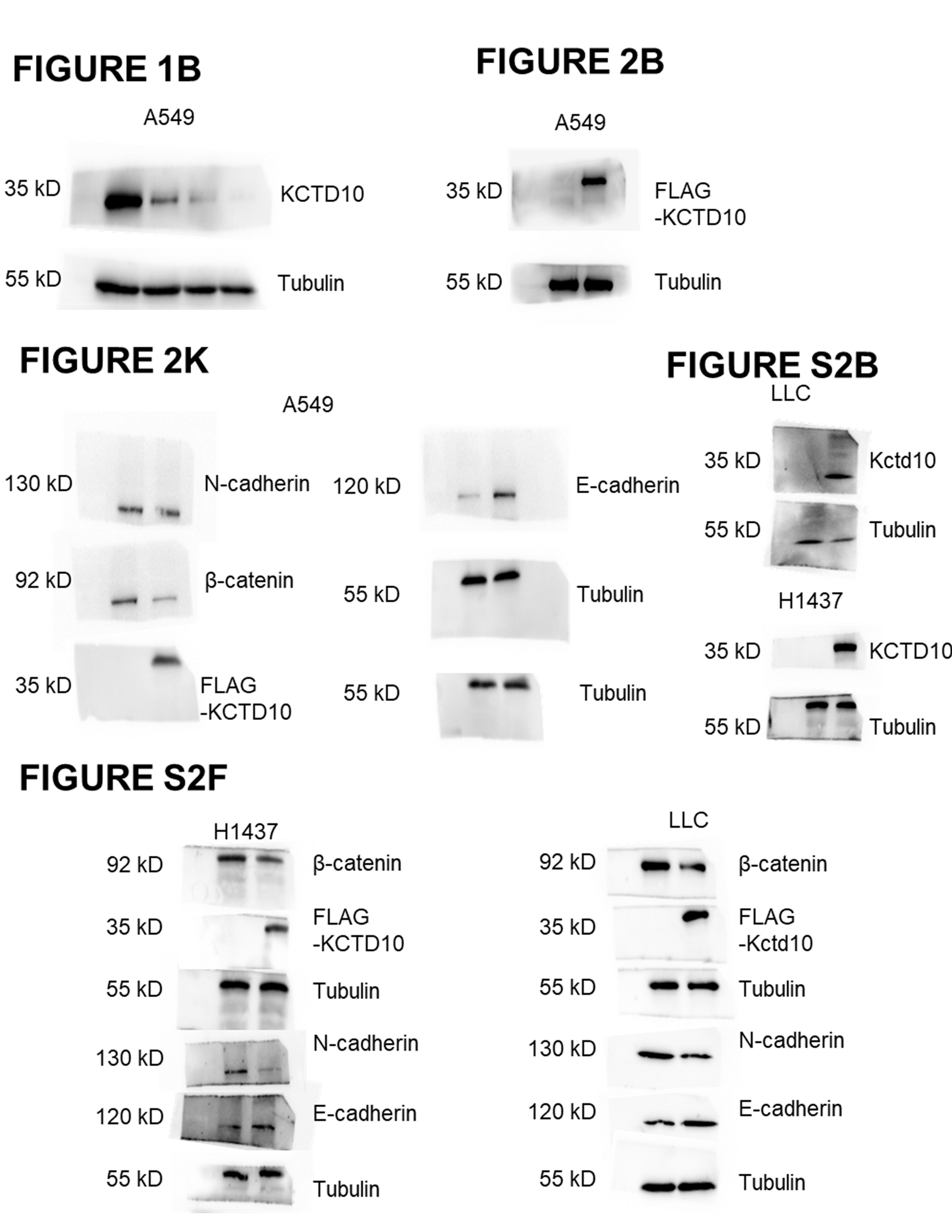

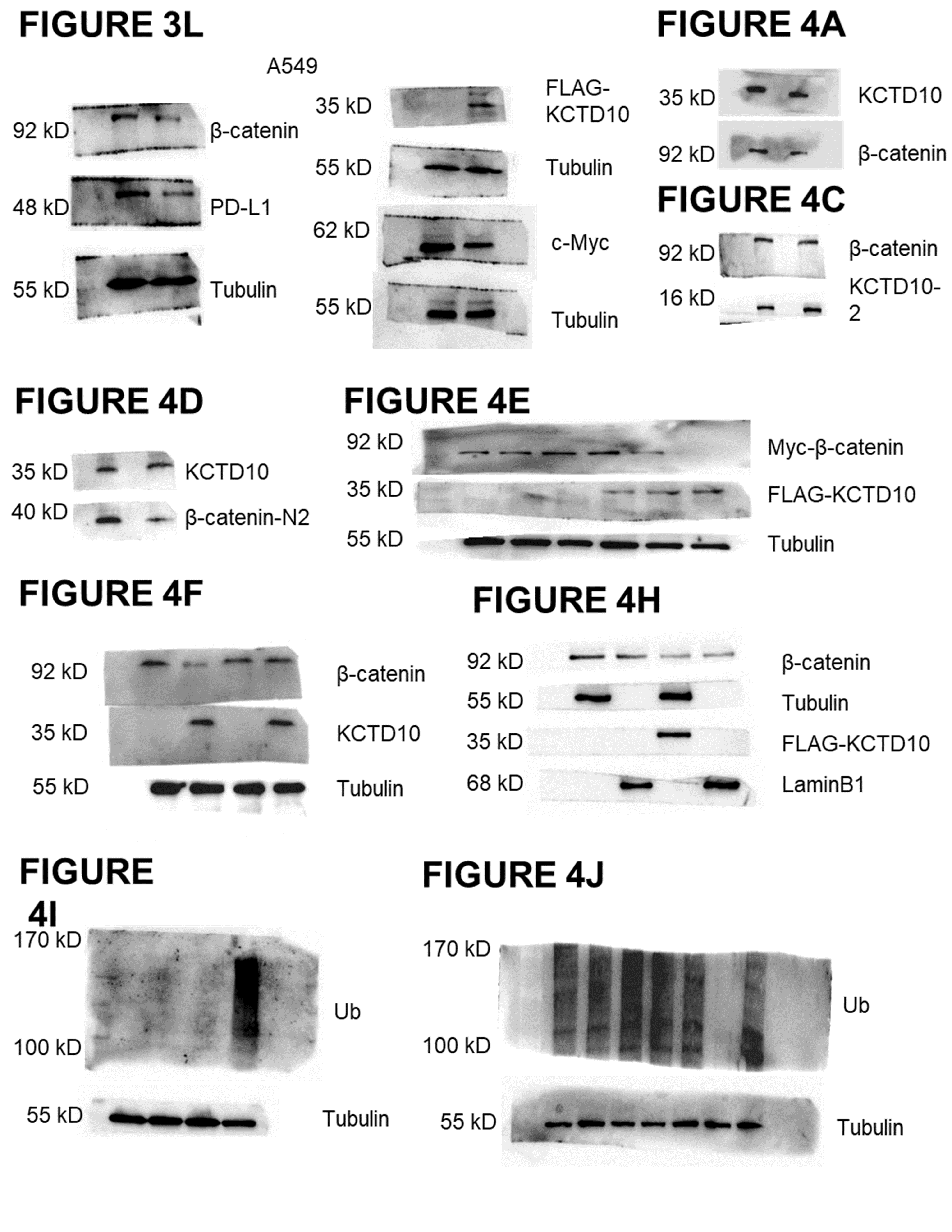

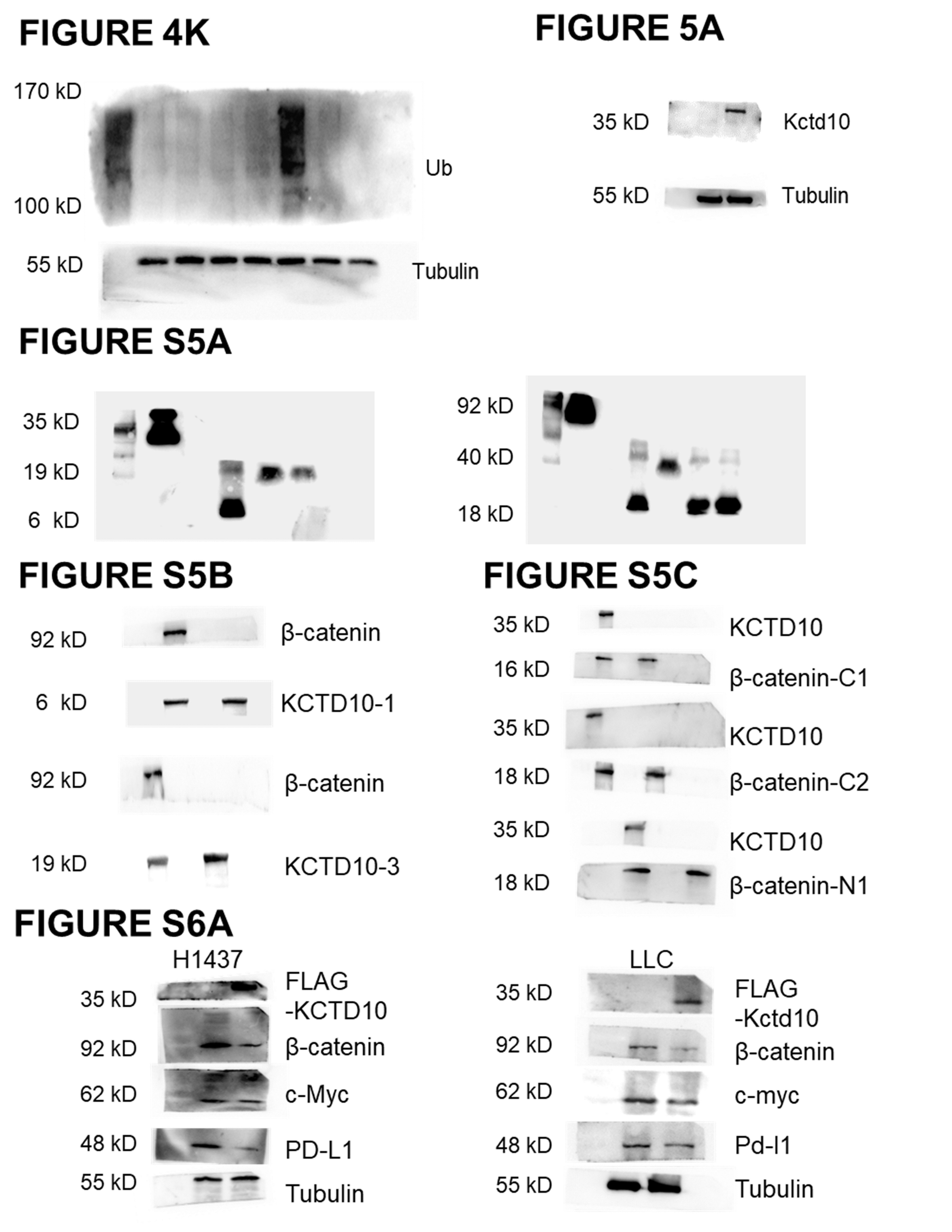

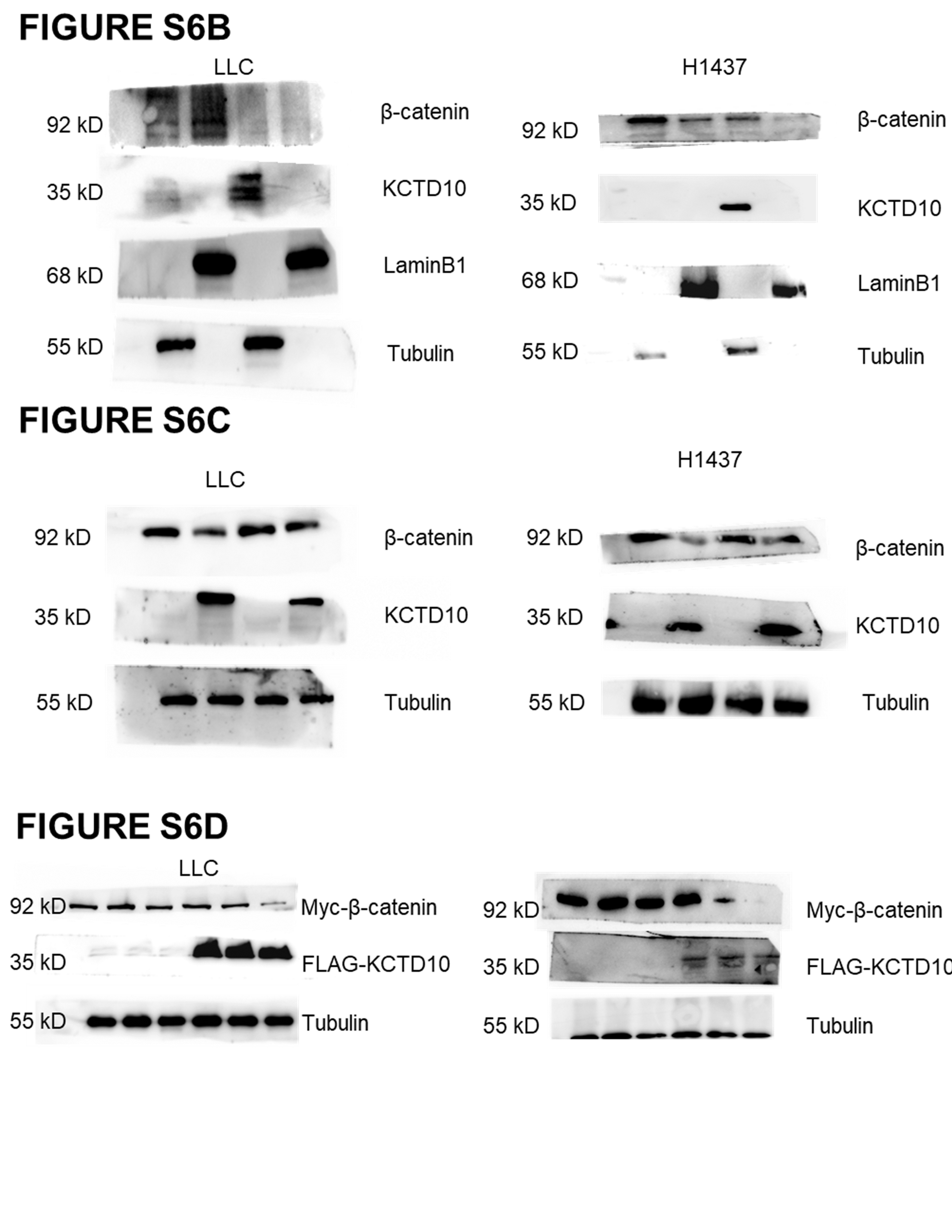

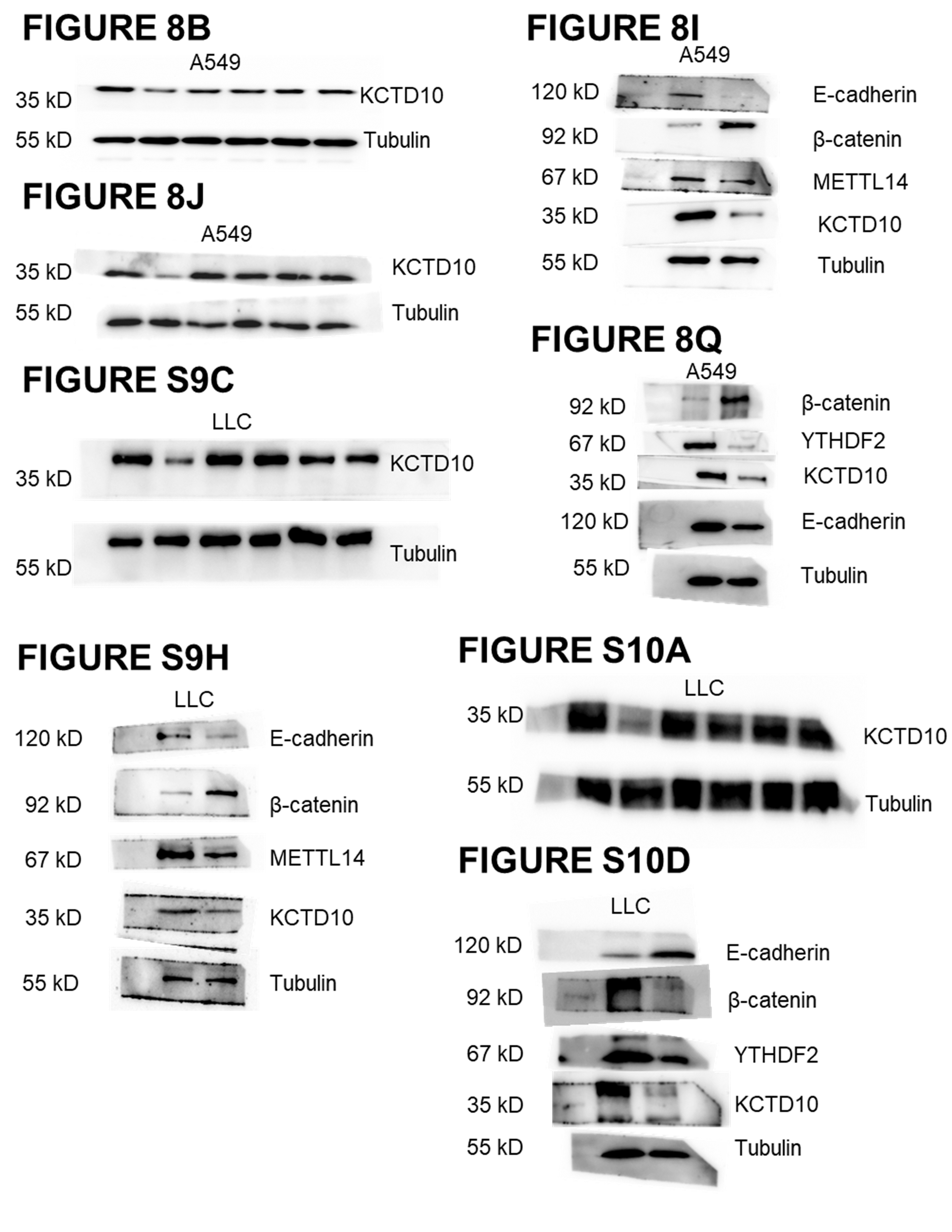

Supplement: Supplementary file 2 [file Table2.docx]
